# The effect of cuproptosis-relevant genes on the immune infiltration and metabolism of gynecological oncology by multiply analysis and experiments validation

**DOI:** 10.1038/s41598-023-45076-5

**Published:** 2023-11-09

**Authors:** Xiao-min Ran, Hui Xiao, Yan-xiang Tang, Xia Jin, Xing Tang, Juan Zhang, Hui Li, Yu-kun Li, Zhen-zi Tang

**Affiliations:** 1 Department of Gynecologic Oncology Ward5, Hunan Cancer Hospital, The Affiliated Cancer Hospital of Xiangya School of Medicine, Central South University, Changsha, Hunan China; 2 Department of Gynecologic Oncology Ward1, Hunan Cancer Hospital, The Affiliated Cancer Hospital of Xiangya School of Medicine, Central South University, Changsha, Hunan China; 3 Department of Pathology, Hunan Cancer Hospital, The Affiliated Cancer Hospital of Xiangya School of Medicine, Central South University, Changsha, Hunan China; 4 Department of Obstetrics and Gynecology, The Second Affiliated Hospital of the University of South China, Hengyang, Hunan China; 5 Department of Assisted Reproductive Centre, Zhuzhou Central Hospital, Xiangya Hospital, Zhuzhou Central South University, Central South University, Zhuzhou, Hunan China

**Keywords:** Cancer, Cell biology

## Abstract

Gynecological cancers are a leading cause of mortality for women, including ovarian cancer (OC), cervical squamous cell carcinoma (CESC), and uterine corpus endometrial carcinoma (UCEC). Nevertheless, these gynecological cancer types have not elucidated the role of cuproptosis and the correlated tumor microenvironment (TME) infiltration features. CRGs had important potential molecular functions and prognostic significance in gynecological cancers, especially in UCEC. Hub CRG, FDX1, was correlated with the CD8+ T cell immune infiltration in UCEC and CESC. FDX1 OE could significantly repress the proliferation ability in UCEC cells by MTT, EdU, and clone formation. High levels of FDX1 could repress ATP and lactic acid but enhance ROS and glucose levels by metabolism assay. The xenograft tumor model indicated that FDX1 OE significantly inhibited the growth of UCEC and attenuated the PCNA, HK2, PKM2, and Ki-67 expression. These CRGs are significant roles that could be potential markers and treatment targets to optimize the TME immune cell infiltration features for gynecological cancer types. FDX1 is a hub CRGs in UCEC to promote immune infiltration and attenuate proliferation and metabolism.

## Introduction

Gynecological cancer mainly includes ovarian cancer (OC), cervical squamous cell carcinoma (CESC) and uterine corpus endometrial carcinoma (UCEC), which are serious malignant diseases in women and have resulted in a serious social burden. Nevertheless, useful diagnostic and prognostic markers for detection and effective therapeutic targets for clinical treatment remain lacking^1^. Moreover, numerous studies indicated that the progression of these cancer types was very similar, which implicated share some common diagnostic and prognostic markers as well as therapeutic targets. For example, progressive neoplastic alterations to the premalignant alterations were observed in both UCEC and CESC^2^. A large sample of transcriptomics found OC, CESC, and UCES had a common pathological basis, such as aberrant DNA replication^3^, estrogen disturbances^4^, HPV infection, and androgen disorder^5^. In carcinogenesis, aberrant transcriptomes occur in essentially all cancer types^6,7^. With the development of whole-genome sequencing, an increase of differentially expressed genes (DEGs) in patients with different cancer types has been identified, suggesting that cancer is a complex malignant disease^8^. For validating the diagnostic and prognostic markers and molecular mechanism correlated with each type of gynecological cancer and the underlying correlation among OC, CESC, and UCEC, the contribution of validated hub DEGs to the physiopathologic mechanism of gynecological cancer must be elucidated.

The copper ion is involved in many biological functions^9^. Various diseases are associated with changes in copper ion homeostasis, including inflammation, neurodegeneration, and cancer^10,11^. Cuproptosis-relevant genes (CRGs) mainly include two parts, one is the lipoic acid pathway (FDX1, LIPT1, LIAS, and DLD), and the other is pyruvate dehydrogenase complex (DLAT, PDHA1, PDHB, MTF1, GLS, and CDKN2A)^12,13^. FDX1, a ferredoxin protein, reduces cytochrome P450 to drive steroid biosynthesis and drug metabolism^14^. As a direct target of elesclomol, this protein can also transform the Cu^2+^ to Cu^1+^ (a more toxic form), which further implies the great potential of cuproptosis in the clinical treatment of tumors^15^. In previous study, FDX1 was increased in cisplatin‑resistant OC cells, and attenuated ferroptosis induced by cisplatin^16^. Chen found that FDX1 was significantly enhanced in UCEC patients^17^. However, the molecular functions of FDX1 was still unclear in UCEC progression.

A tumor microenvironment comprises signaling molecules and stroma^18^, especially in OC, CESC, and UCSC^19,20^. A major influence on immune monitoring is the discovery that metabolic considerations and signaling pathway modulation affect immune function^21^. However, the role of cuproptosis has not been definitively linked to the development of gynecological cancer, particularly OC, CESC, and UCEC.

In this study, we devised a series of bioinformatics analyses and experimental validation to elucidate the potential role and possible molecular mechanisms of cuproptosis in these gynecological tumors, especially its effect on immune infiltration. The expression and DNA alteration profiles of these CRGs were confirmed by TCGA and the cBioProtal database. PPI networks were further constructed by the GenMANIA database. Molecular subtypes for these gynecological cancers further suggested the potential effect of cuproptosis on the multiple molecular phenotypes. Establishing prognostic indicators were constructed by these PPI network hub genes, demonstrating their prognostic value. Moreover, we confirmed the effect of FDX1 on CD8^+^ cell infiltration in OC, CESC, or UCEC patients. Finally, our study determined that FDX1 could impede the development and progression of UCEC by attenuating the Akt pathway.

## Material and methods

Please see the Supplementary data 1.

## Results

### The mRNA level of CRGs in OC, CESC, and UCEC

**Figure 1:**
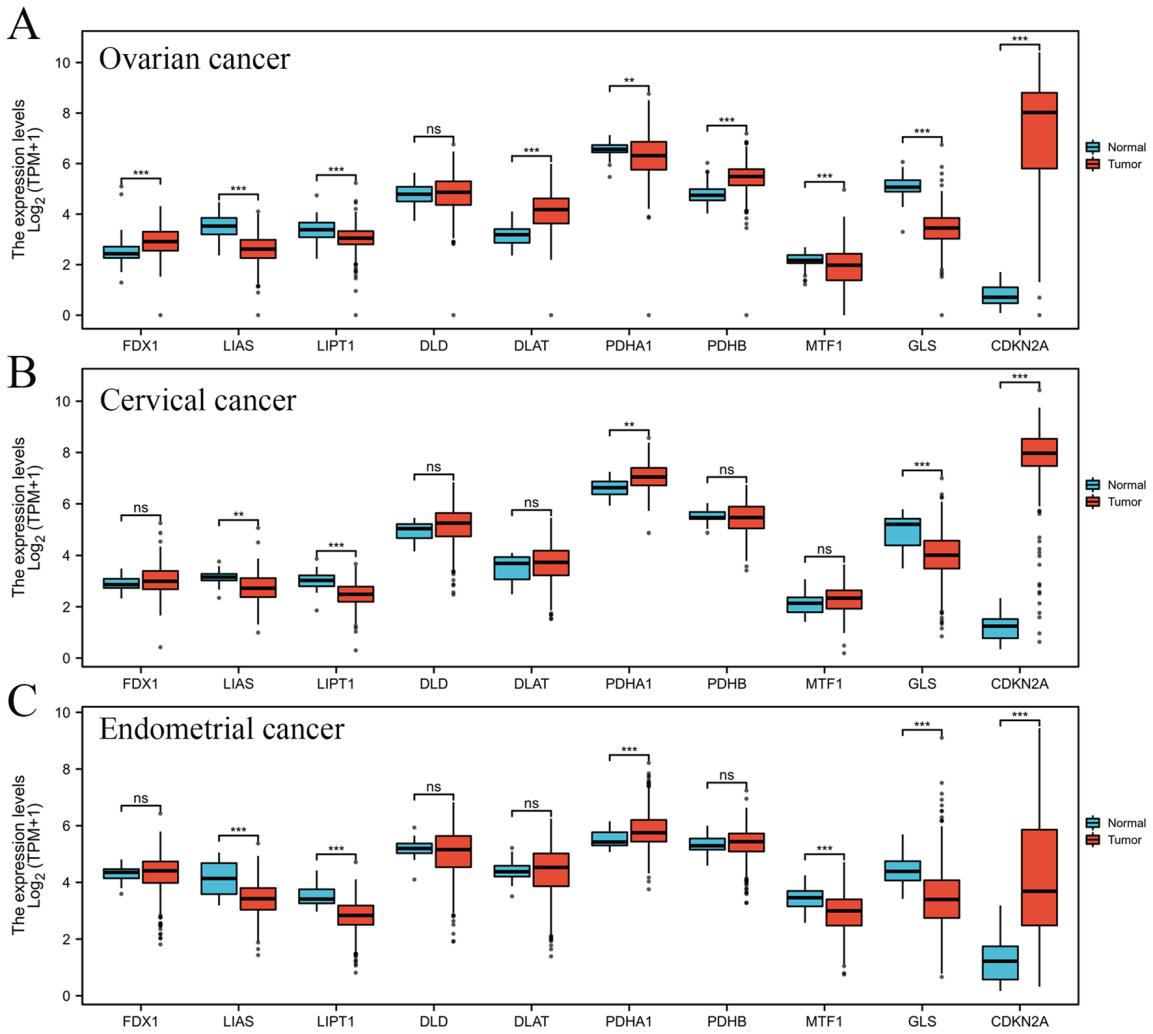
The expression of CRGs in different gynecological cancer types. CRGs mRNA expression in (**A**) OC, (**B**) CESC, and (**C**) CESC. *P < 0.05, **P < 0.01 and ***P < 0.001.

TCGA database was utilized to confirm CRGs level in OC, CESC, UCEC, and corresponding para-carcinoma tissues (Fig. 1). We found that the lipoic acid (LA) pathway genes of LIAS and LIPT1 were significantly decreased in both gynecological oncology types. FDX1 was only obviously increased in OC, and the levels of DLD were not significantly changed in all these gynecological oncology types. PDH complex includes two parts: DLAT, PDHA1, and PDHB for promoting cuproptosis and MTF1, GLS and CNKN2A for inhibiting cuproptosis. These PDH mRNAs were both dysregulated in OC. DLAT, PDHB, and MTF1 were not changed in CESC. The levels of DLAT and PDHB were not significantly changed in UCEC.

Moreover, we also confirmed the DNA alteration of CRGs in these gynecological cancers (Supplementary Fig. 1), which showed that OC has an obvious DNA alteration level in these CRGs, especially in DNA amplification. The DNA alteration of CRGs was primarily enriched in DNA deletion types in CESC patients. The missense mutation was the most common mutation type in UCEC patients. These results indicated that these gynecological oncology types responded with differing degrees of cuproptosis dysregulation, indicating that cuproptosis might be an important research orientation in clinical diagnosis, prognosis, and treatment for patients with gynecological oncology.

### Function enrichment of CRGs in different gynecological oncology types

**Figure 2:**
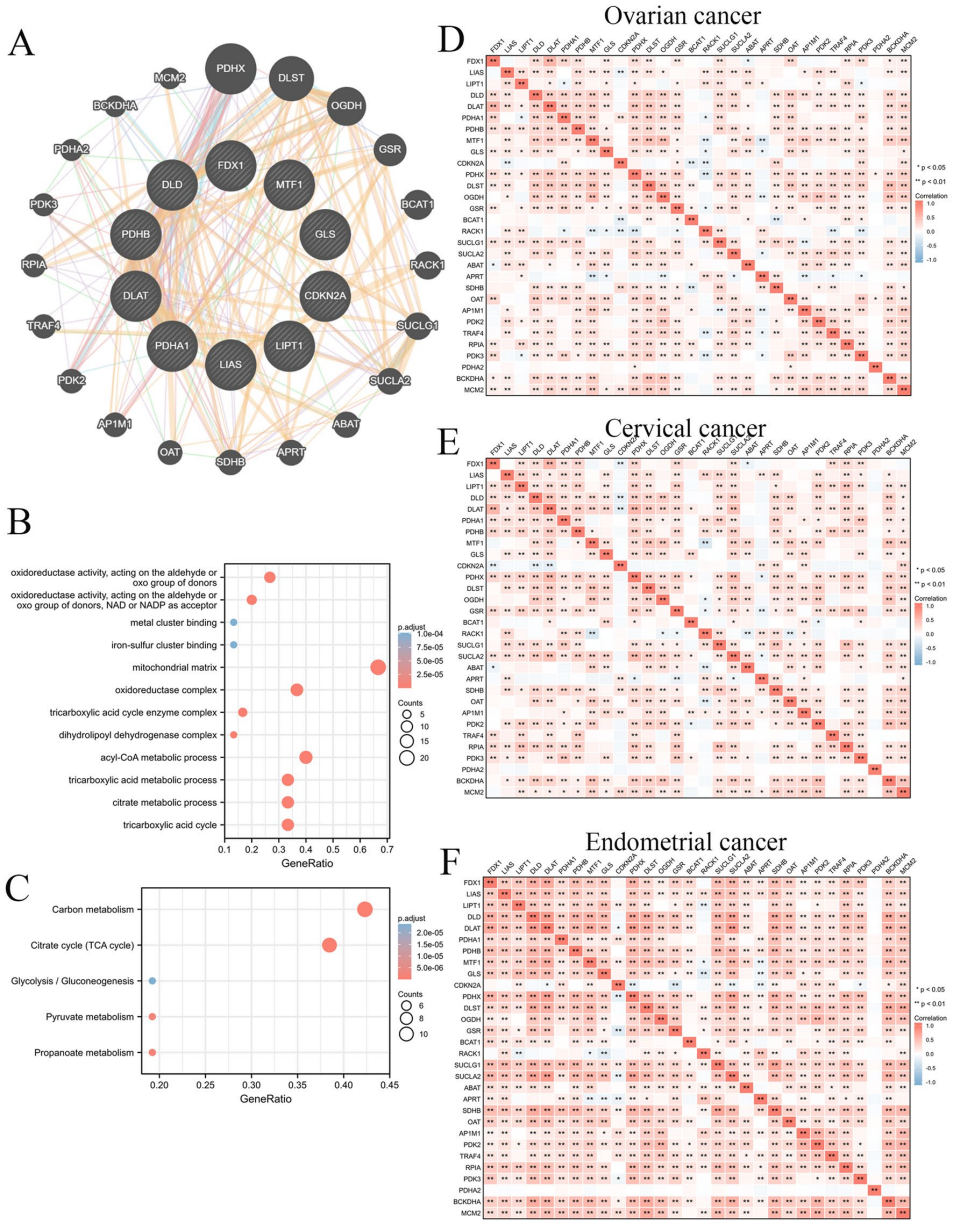
Co-expression, functions, and interactions with CRGs. (**A**) The PPI network among the CRGs is based on the GeneMANIA database. (**B**) GO function enrichment of PPI network genes. (**C**) KEGG pathway enrichment of PPI network genes. (**D**) The co-expression of these PPI network genes in OC. (**E**) The co-expression of these PPI network genes in CESC. (**F**) The co-expression of these PPI network genes in UCEC.

Next, we constructed a CRGs network by GeneMANIA database, including 30 genes (Fig. 2A). These CRGs might interact with a series of genes, including PDHX, DLST, OGDH, GSR, BCAT1, RACK1, SUCLG1, SUCLA2, ABAT, APRT, SDHB, OAT, AP1M1, PDK2, TRAF4, RPIA, PDK3, PDHA2, BCKDHA, and MCM2. We further confirmed the enrichment for these genes by the DAVID database. In GO enrichment terms, these genes were significantly enriched in metal cluster binding, iron-sulfur cluster binding, mitochondrial matrix, oxidoreductase complex, and tricarboxylic acid cycle enzyme complex (Fig. 2B). Regarding KEGG enrichment terms, these genes involved carbon metabolism, the citrate cycle, and glycolysis/gluconeogenesis (Fig. 2C). Moreover, correlation analysis further indicated that these genes had significant associations between the expression of interacted genes with each other in OC, CESC, and UCEC (Fig. 2D–F). These results suggested that the CRGs involved multiple metabolism progressions for OC, CESC, and UCEC, especially in the TCA cycle.

### Classification of each cuproptosis-related phenotype for gynecological oncology

We used the R package of ConsensusClusterPlus to classify OC patients into clusters (cluster1 and 2) (Supplementary Fig. 2A). These differentially expressed genes (DEGs) (Supplementary Fig. 2B,C) were significantly enriched in proteoglycans in cancer, Hippo signaling pathway, hedgehog signaling pathway, and apelin signaling pathway for KEGG term, and regulation of DNA metabolic process, negative regulation of hippo signaling, and epithelial to mesenchymal transition (Supplementary Fig. 2D,E). Moreover, these ferroptosis-related signatures were significantly changed (Supplementary Fig. 3A), indicating a potential correlation between cuproptosis and ferroptosis. The expression level distribution of immune scores in clusters 1 and 2 indicated that the percentage abundance of tumor-infiltrating immune cells (T cell CD4^+^, endothelial cell, and T cell CD8^+^) was significantly decreased in cluster 1, but Macrophage and NK cell was increased (Supplementary Fig. 3B,C). Moreover, the immune checkpoint, SIGLEC15, was significantly decreased, and other immune checkpoints (HAVCR2, PDCD1LG2, and CD274) were significantly increased in cluster 1 (Supplementary Fig. 3D). Nevertheless, correlation analysis of OCLR score analysis indicated that it was not significant between clusters 1 and 2 (Supplementary Fig. 3E). Drug-sensitive analysis showed that the effects of paclitaxel and docetaxel were more sensitive in cluster 1 than in cluster 2 (Supplementary Fig. 3F).

Homoplastically, we classify CESC patients into two clusters based on the PPI network 30 genes (Supplementary Fig. 4A). The significant DEGs between clusters 1 and 2 (Supplementary Fig. 4B,C) were enriched in ubiquitin-mediated proteolysis, pyrimidine metabolism, nicotinate and nicotinamide metabolism, and estrogen signaling pathway for KEGG (UP) term (Supplementary Fig. 4D), and sphingolipid metabolism, retinol metabolism, pyruvate metabolism, hippo signaling pathway, glutathione metabolism, ferroptosis, and drug metabolism-cytochrome P450 for KEGG (DOWN) term (Supplementary Fig. 4E). These DEGs were also enriched in zinc ion homeostasis, sequestering of metal ions, and humoral immune response for GO (UP) term (Supplementary Fig. 4F), and urogenital system development, ureteric bud development and respiratory tube development for GO (DOWN) term (Supplementary Fig. 4G). We also found a close correlation between cuproptosis and ferroptosis in clusters 1 and 2 (Supplementary Fig. 5A). The expression level distribution of immune scores in clusters 1 and 2 indicated that the percentage abundance of tumor-infiltrating immune cells (T cell CD4^+^, endothelial cell, and T cell CD8^+^) was significantly decreased in cluster 1, but Macrophage and NK cell were significantly opposite (Supplementary Fig. 5B,C). Furthermore, the immune checkpoints (SIGLEC15, LAG3, PDCD1LG2, and CD274) were significantly decreased in cluster 1 (Supplementary Fig. 5D). Nevertheless, correlation analysis of OCLR score analysis indicated that it was not significant between clusters 1 and 2 (Supplementary Fig. 5E). Drug-sensitive analysis showed that the effects of paclitaxel and docetaxel were more sensitive in cluster 1 than in cluster 2 (Supplementary Fig. 5F).

Similarly, we classify UCEC patients into two clusters by a similar approach (Supplementary Fig. 6A). These significant DEGs between clusters 1 and 2 (Supplementary Fig. 6B,C) were enriched in the Wnt signaling pathway, Ras signaling pathway, Hippo signaling pathway, and glycosaminoglycan degradation for KEGG (UP) term (Supplementary Fig. 6D), and propanoate metabolism, platinum drug resistance, Hippo signaling pathway, cell cycle, and autophagy (Supplementary Fig. 6E). These DEGs were also enriched in the regulation of the collagen metabolic process and glycoprotein metabolic process for GO (UP) term (Supplementary Fig. 6F), and proteasomal protein catabolic process, centrosome cycle, and DNA replication for GO (DOWN) term (Supplementary Fig. 6G). We also found a close correlation between cuproptosis and ferroptosis in clusters 1 and 2 for UCEC patients (Supplementary Fig. 7A). The immune score indicated that the percentage abundance of tumor-infiltrating immune cells (endothelial cell, B cell, Macrophage, NK cell, and T cell CD8^+^) was significantly decreased in cluster 1, but T cell CD4^+^ were significantly opposite (Supplementary Fig. 7B,C). Furthermore, the immune checkpoint, PDCD1, was significantly decreased, and other immune checkpoints (CD274, PDCD1LG2, HAVCR2, and TIGIT) were significantly increased in cluster 1 (Supplementary Fig. 7D). Interestedly, the correlation analysis of OCLR score analysis indicated that it was significantly increased in cluster 1 (Supplementary Fig. 7E). Drug-sensitive assay suggested cisplatin and docetaxel were more sensitive in cluster 1, and the effects of paclitaxel were more sensitive in cluster 2 than cluster 1 (Supplementary Fig. 7F).

### Evaluation of clinical outcomes in OC, CESC, and UCEC patients based on the Cuproptosis network genes

For confirming the prognostic values of CRGs, we used 30 cuproptosis network genes to predict prognostic significance for OC, CESC, and UCEC patients based on multi-factor Cox regression and the step function performed the iteration^22^. Firstly, 5 key genes were closely associated with the prognosis of OC patients: CDKN2A, PDHX, SUCLA2, SDHB, and OAT (Supplementary Fig. 8A). The Riskscore= (− 0.0607)* CDKN2A+ (− 0.3064)* PDHX+ (0.2156)* SUCLA2+ (0.2532)* SDHB+ (0.1247)* OAT. These OC patients were divided into high-risk or low-risk groups with the risk score based on the TCGA database OC dataset. OC patients' survival time and rate were significantly reduced, with the risk score enhanced (Supplementary Fig. 8B). The AUCs of the ROC curve at 1, 3, and 5 years were 0.58, 0.59, and 0.634, respectively (Supplementary Fig. 8C). Moreover, we also found that the expression of CD4^+^ cells, CD8^+^ cells, endothelial cells, and NK cells were significantly reduced with the risk score enhanced (Supplementary Fig. 8D), which indicated that immune infiltration played an important role in the cuproptosis-related prognostic models for OC patients.

Next, we used a similar way to construct a prognostic model for CESC patients. 7 signatures were associated with CESC prognosis: FDX1, PDHA1, DLST, SUCLA2, PDK2, BCKDHA and MCM2 (Supplementary Fig. 9A). The Riskscore= (− 0.4377)* FDX1+ (− 0.5309)* PDHA1+ (0.4468)* DLST+ (0.5285)* SUCLA2+ (−0.6105)* PDK2+ (0.4047)* BCKDHA+ (− 0.4118)* MCM2. The overall survival analysis showed that the survival time and rate of CESC patients were significantly reduced with the risk score enhanced (Supplementary Fig. 9B). The AUCs of the ROC curve at 1, 3, and 5 years were 0.62, 0.73, and 0.76, respectively (Supplementary Fig. 9C). Furthermore, B cell was reduced with the risk score enhanced, but CD4^+^ cell and endothelial cell were positively correlated with the risk score (Supplementary Fig. 9D), which indicated that the CD4^+^ cell immune infiltration might be an opposite effect role in cuproptosis-related prognostic models for CESC patients compared to OC patients.

Then, we constructed a prognostic model for UCEC patients. 5 key signatures were associated with the UCEC prognosis: GLS, CDKN2A, PDHX, SUCLG1, and TRAF4 (Supplementary Fig. 10A). The Riskscore= (0.2899)* GLS+ (0.1578)* CDKN2A+ (− 0.3782)* PDHX+ (0.8055)* SUCLG1+ (0.2636)* TRAF4. The overall survival analysis showed that the survival time and rate of UCEC patients were significantly reduced, with the risk score enhanced (Supplementary Fig. 10B). The AUCs of the ROC curve at 1, 3, and 5 years were 0.597, 0.728, and 0.72, respectively (Supplementary Fig. 10C). Furthermore, we also found the B cell and CD4^+^ cell was significantly reduced with the risk score enhanced (Supplementary Fig. 10D), which indicated that the B cell and CD4^+^ cell immune infiltration might play key roles in cuproptosis-related prognostic models for UCEC patients.

### Drug sensitivity assay for CRGs

We used drug sensitivity analysis to confirm the clinical value of hub CRGs, as shown in Supplementary Fig. 11A. Only a few drugs could regulate FDX1 expression. Therefore, we selected FDX1 as the key CRGs for further study.

### The molecular and clinical characteristics of FDX1 in gynecological oncology

**Figure 3:**
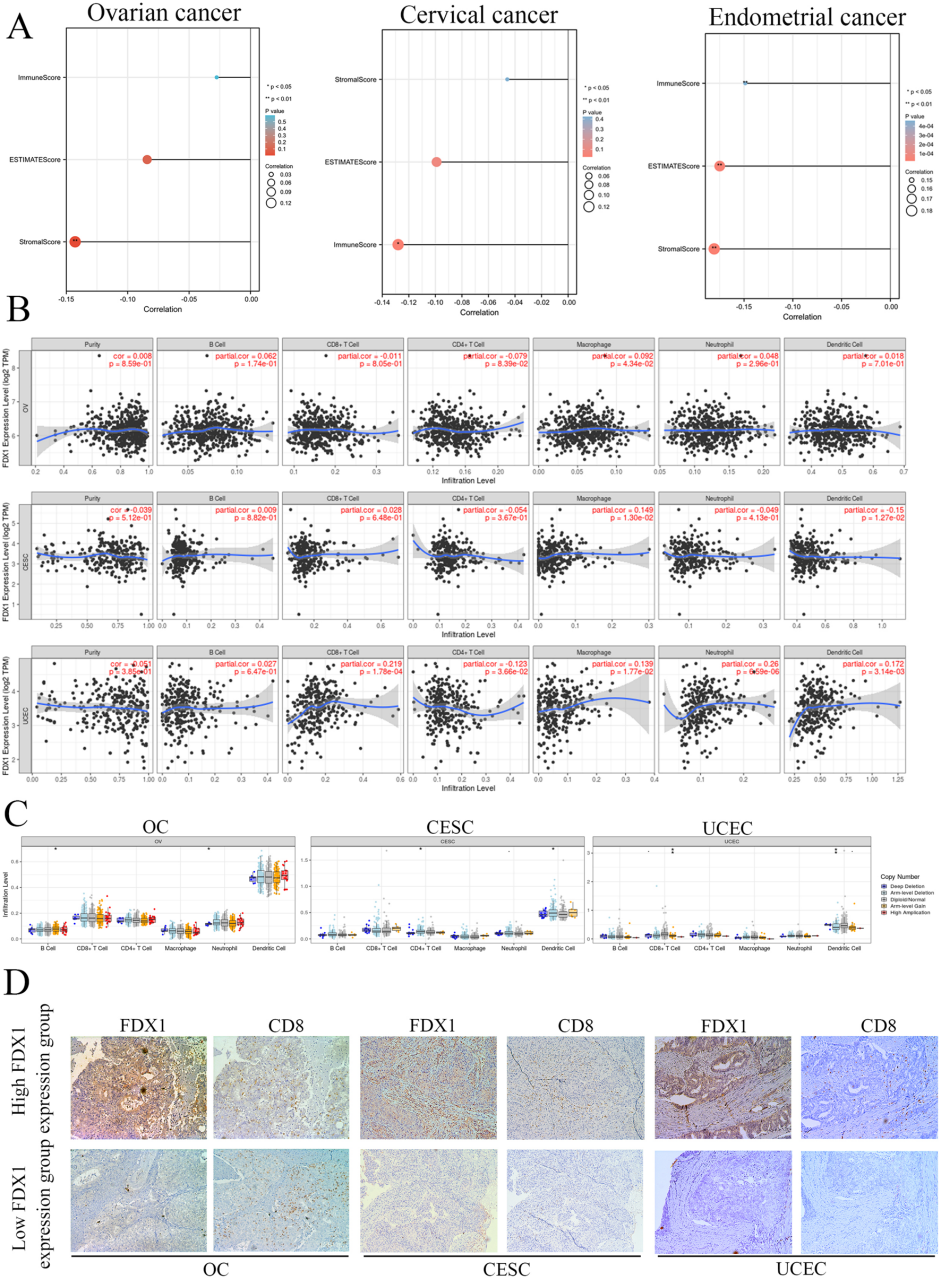
The correlation between FDX1 expression and immune infiltration in different gynecological cancer types. (**A**) The correlation of FDX1 with an immune score, ESTIMATE score, and stromal score in OC, CESC, and UCEC by ESTIMATE algorithm. (**B**) The correlation of FDX1 expression with different immune cell infiltration in OC, CESC, and UCEC by TIMER algorithm. (**C**) The CNV affects the distribution of FDX1 in OC, CESC, and UCEC. (**D**) CD8^+^ T cell infiltration in OC, CESC, and UCEC by IHC staining. *P < 0.05, **P < 0.01 and ***P < 0.001.

To validate the molecular and clinical features of FDX1 in gynecological oncology, we used GSEA analysis to preliminarily predict the molecular function of FDX1 in OC, CESC, and UCEC. We found FDX1 was significantly correlated with Notch expression and processing, PD1 signaling, and mitochondria pathway in OC patients (Supplementary Fig. 11B). In CESC patients, FDX1 was involved in the downstream signaling of activated FGFR4, diseases of metabolism, and SHC-mediated cascade FGFR4 (Supplementary Fig. 11C). For UCEC patients, FDX1 played a key role in mitochondrial gene expression and arachidonic acid metabolism (Supplementary Fig. 11D). We also confirmed the association between FDX1 and clinicopathological parameters for OC, CESC, and UCEC patients by TCGA (Supplementary Tables 1–3). FDX1 was associated with age in OC patients, with T stage, N stage, histological type, and histologic grade in CESC patients, with histologic grade and surgical approach for UCEC patients. Moreover, we used the R package of Estimate to analyze immune infiltration score in OC, CESC, and UCEC patients, which indicated that the FDX1 was correlated with a stromal score in OC, with an immune score in CESC, and with an immune score, estimate score and immune score in UCEC based on TCGA database (Fig. 3A). FDX1 mRNA level was correlated with CD8^+^ T cells in UCEC patients using the TIMER database (Fig. 3B). FDX1 DNA alteration was associated with CD8^+^ T cells in UCEC patients (Fig. 3C). Finally, we used IHC staining to confirm CD8^+^ T cell infiltration between high and low FDX1 expression in OC, CESC, and UCEC patients. The result showed that FDX1 was significantly correlated with CD8^+^ T cell infiltration in CESC and UCEC patients (Fig. 3D). Taken together, the protein and mRNA levels of FDX1 was significantly correlated with immune infiltration in UCEC.

### FDX1 could significantly repress cell proliferation and glycolysis in vitro

**Figure 4:**
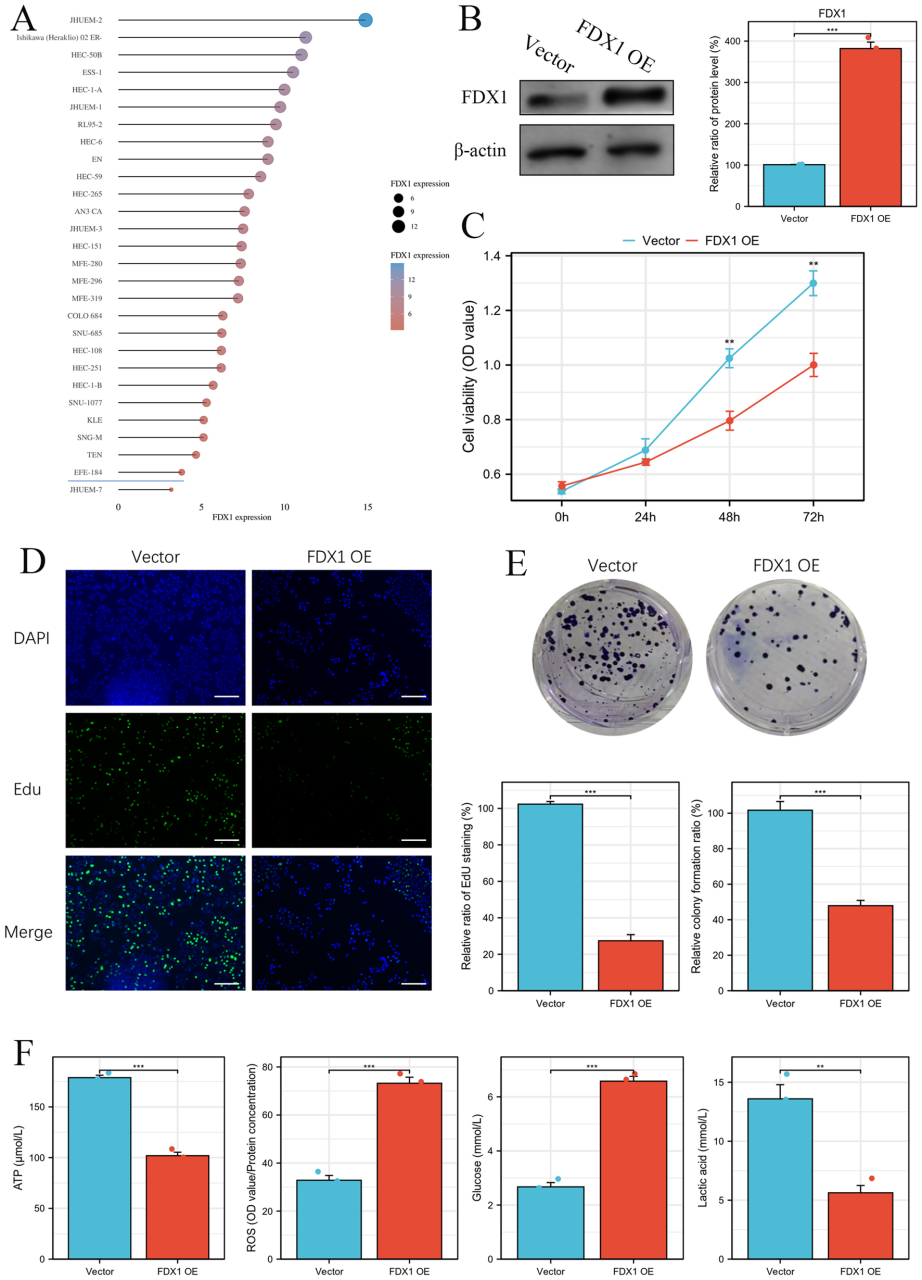
FDX1 inhibits UCEC cell proliferation and metabolism in vitro. (**A**) FDX1 expression in UCEC cell lines by CCLE database. (**B**) Validation of FDX1 expression in UCEC cell transfected with FDX1 OE or vector by western blot. The effect of FDX1 OE on UCEC cell viability by MTT (**C**), EdU (**D**), and clone formation (**E**). (**F**) The effect of FDX1 OE on UCEC cell metabolism, including ATP, ROS, Glucose, and Lactic acids. **P < 0.01 and ***P < 0.001.

FDX1 expression in UCEC cell lines was analyzed by the CCLE database (Fig. 4A). We constructed FDX1 overexpressed UCEC cells and validated them by Western blot (Fig. 4B). FDX1 overexpression could repress the cell viability compared to UCEC cells transfected with vector plasmid by MTT (Fig. 4C). FDX1 could inhibit proliferation for UCEC cells by EdU staining (Fig. 4D). Clone formation assay showed that the proliferation ability of UCEC cells was significantly decreased in the FDX1 OE group compared to the vector group (Fig. 4E). Moreover, the ATP and the lactic acid level was decreased in the FDX1 OE group, but the levels of ROS and Glucose was significantly increased in the FDX1 OE group compared to the vector group (Fig. 4F), which indicated that FDX1 could significantly repress UCEC cell glycolysis to inhibit cell proliferation.

### The effect of FDX1 OE on UCEC growth and metabolism

**Figure 5:**
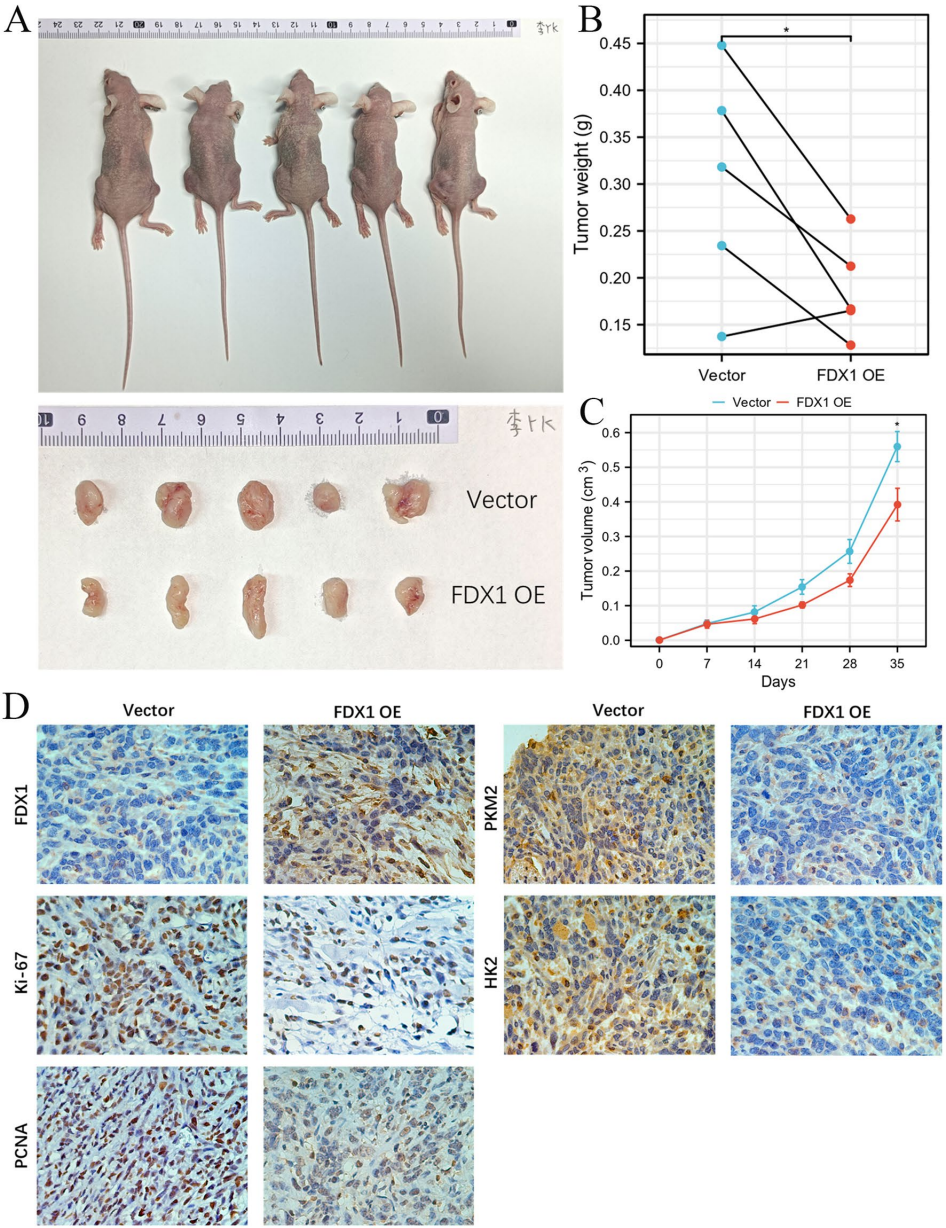
FDX1 inhibits UCEC cell growth and metabolism in vivo. (**A**) The size of xenografts in FDX1 OE and vector group. (**B**) The tumor weight of xenografts in FDX1 OE and vector group. (**C**) The tumor volume of xenografts in FDX1 OE and vector group. (**D**) The expression of FDX1, Ki-67, PCNA, PKM2, and HK2 in xenografts by IHC staining. *P < 0.05.

Furthermore, we found that FDX1 could inhibit UCEC cell growth in vivo (Fig. 5A). The weight and volume of xenografts were decreased in FDX1 OE compared to the vector group (Fig. 5B,C). IHC staining showed that the expression of Ki-67, PCNA, PKM2, and HK2 was significantly decreased along with FDX1 overexpression (Fig. 5D), indicating FDX1 could inhibit UCEC growth by impeding metabolism in xenograft.

### FDX1 driving Akt pathway to inhibit UCEC cell proliferation, migration, and invasion

**Figure 6:**
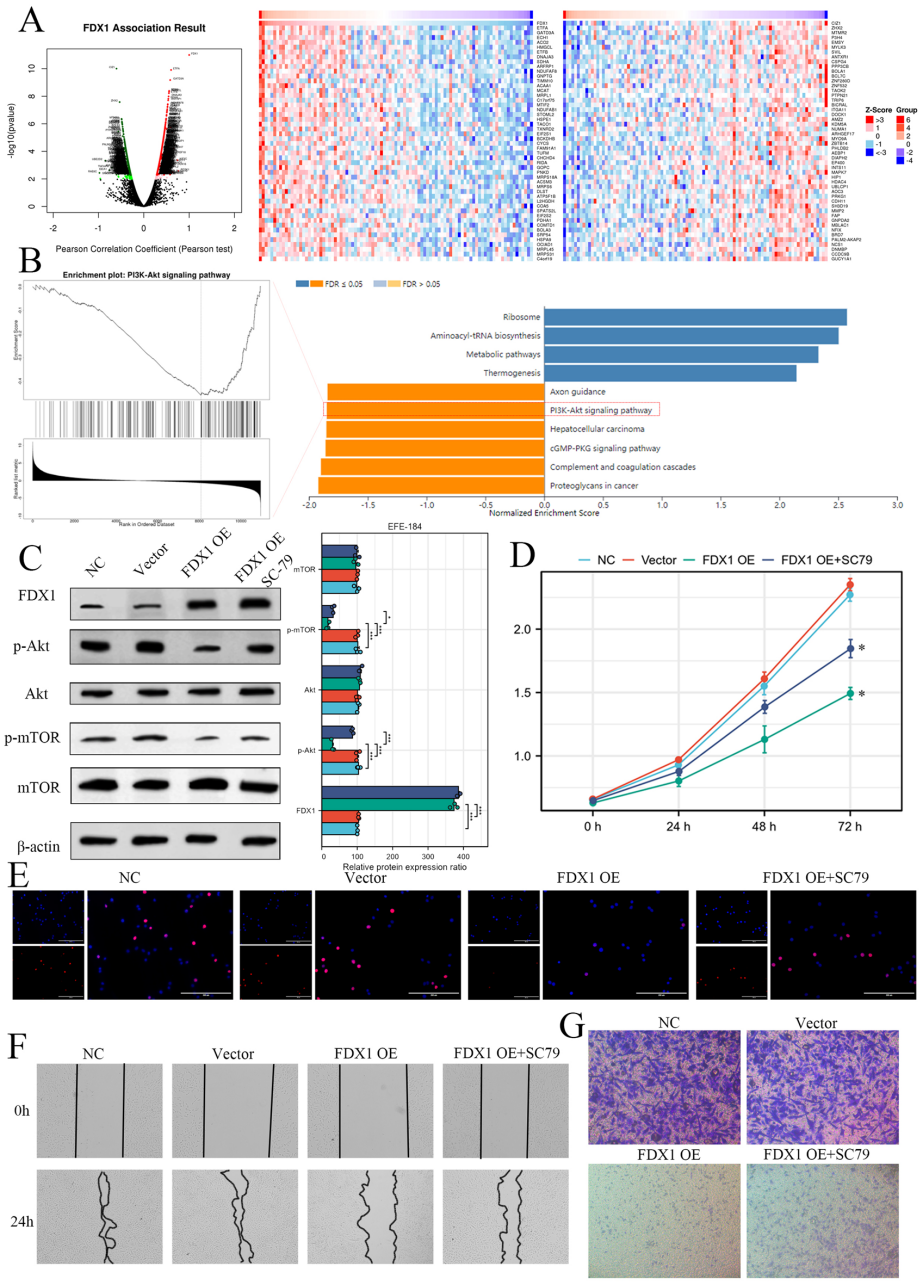
FDX1 inhibits UCEC cell proliferation and migration by the Akt pathway in vivo. (**A**) The FDX1-correlated genes in UCEC by Volcanic plot and heat map. (**B**) The GSEA analysis for these significantly FDX1 correlated genes. (**C**) The effect of FDX1 on the activity of Akt pathway by western blot. (**D**) MTT analysis for the role of FDX1 OE and FDX1 OE plus Akt activating agent in UCEC cell. (**E**) Edu staining analysis for confirming FXD1-Akt axis in UCEC proliferation ability. The migration and invasion ability of UCEC cells transfected with FDX1 OE and co-cultured with SC-79 by wound healing assay (**F**) and transwell invasion assay (**G**).

Finally, we used the Linkedomics database (http://www.linkedomics.org/login.php) to confirm the significantly FDX1 correlated genes in UCEC patients (Fig. 6A). GSEA analysis for these correlated genes showed that FDX1 might be impeded PI3K-Akt signaling pathway (Fig. 6B). Western blot showed that FDX1 OE could inactivate the Akt pathway, which could be rescued by the Akt activator (SC-79) (Fig. 6C). MTT and EdU analysis showed that FDX1 OE impeded cell proliferation, which could be rescued by SC-79 (Fig. 6D,E). FDX1 OE inhibits the UCEC cell migration, and SC-79 could attenuate the effect of FDX1 OE on UCEC migration by wound healing assay (Fig. 6F). Transwell invasion assay showed that FDX1 OE inhibits UCEC cell invasion, which was rescued by Akt activator SC-79 (Fig. 6G).

### FDX1 promote immune cell infiltration in UCEC

**Figure 7:**
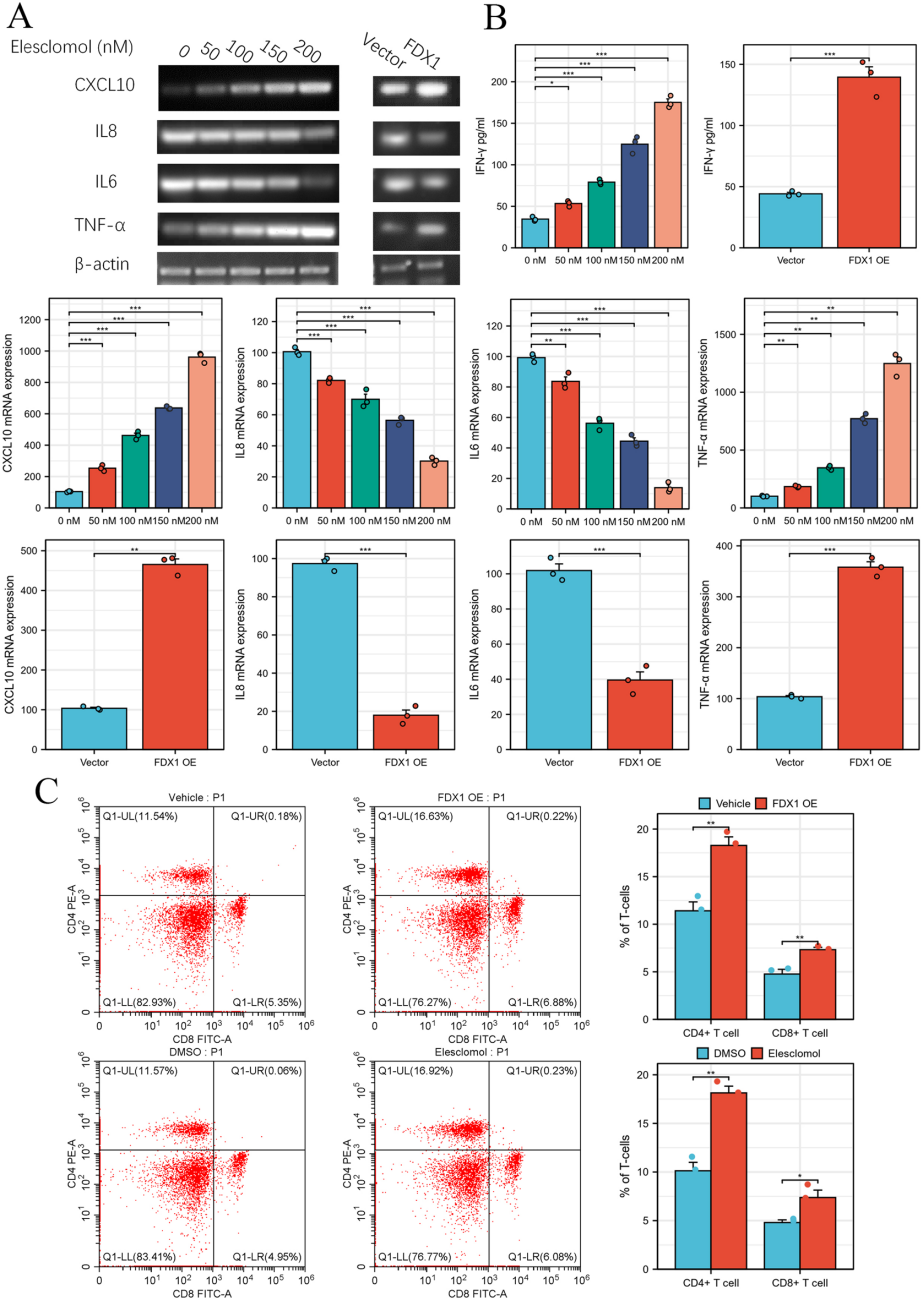
Elesclomol, a FDX1 activator, promote UCEC immune infiltration. (**A**) The effects of elesclomol and FDX1 OE on CXCL10, TNF-α, IL-6 and IL-8 mRNA in UCEC cells. (**B**) The levels of IFN-γ in UCEC cell by ELISA analysis. (**C**) The percentage of CD8^+^ and CD4^+^ T cells in PBMC cell co-cultured with UCEC with elesclomol, FDX1 overexpression or not.

Finally, we measured the effect of FDX1 on immune infiltration related markers, including CXCL10, IL-8, IL-6 and TNF-α. PCR analysis showed that elesclomol, a FDX1 activator, could promote CXCL10 and TNF-α, but inhibit IL-8 and IL-6 in a concentration-dependent manner in UCEC cell (Fig. 7A). ELISA analysis showed that IFN-γ could be significantly increased in FDX1 activator and OE group compared to vector group or DMSO group (Fig. 7B). Flow cytometry analysis showed that the CD8+ and CD4+ percentage was obviously enhanced by FDX1 OE or elesclomol compared to vehicle group (Fig. 7C).

## Discussion

Cuproptosis is a novel molecular form of regulated cell death featured by the accumulation of copper ions directly binding to lipo-acylated components of the TCA in mitochondrial respiration^12^. However, the diagnostic, prognostic, and therapeutic significance of gynecological oncology remains unclear. In our study, we found that cuproptosis was significantly correlated with multiple cell metabolism types, including acyl-CoA metabolic process, tricarboxylic acid metabolic process, citrate metabolic process, carbon metabolism, pyruvate metabolism, and propanoate metabolism. Previous studies indicated that dysregulation of tricarboxylic acid and pyruvate metabolism were key in immune cell infiltration for cancer progression^23,24^. To further elucidate the effect of cuproptosis on the progression of gynecological oncology, integrated bioinformatics analysis of CRGs indicating heterogeneous expressions among OC, CESC, and UCEC patients were confirmed, respectively. Validating the different molecular subtypes classified by cuproptosis could enhance our understanding of the molecular role of cuproptosis and guide effective treatment for these gynecological oncology patients.

The CRGs contained two types, which were activated cuproptosis genes (FDX1, LIAS, LIPT1, DLD, DLAT, PDHA1, and PDHB) and inactivated cuproptosis genes (MTF1, GLS, and CDKN2A)^12^. We first confirmed the expression level of 10 CRGs in OC, CESC, and UCEC patients based on the TCGA database. The activated cuproptosis genes (LIAS and LIPT1) were down-regulated, and inactivated cuproptosis genes (GLS and CDKN2A) were upregulated in both OC, CESC, and UCEC patients (Fig. 1). Moreover, the DNA alteration of these CRGs was observed in in OC, CESC, and UCEC patients based on the cBioProtal database (Supplementary Fig. 1), which partially explains the dysregulation of CRG in gynecologic tumors. Nathan et al. found that LIAS could promote the hydroxylation of HIF1α to reduce its activity in tumorigenesis^25^. Ralph J DeBerardinis and his colleagues found that LIPT1 could upregulate 2-ketoacid dehydrogenases related to the TCA cycle to accelerate TCA metabolism^26^. GLS inhibition could induce replication stress to augment chemotherapy sensitivity in OC cells^27^. Estrogen could activate c-Myc to increase the expression of GLS, promoting glutamine metabolism and proliferation in UCEC^28^. PD-0332991, a selective inhibitor of the CDK4/6 kinases, could inhibit cell proliferation, cell cycle, and apoptosis escaping in OC cells, especially in OC cell types with low expression of CDKN2A^29^. These results indicated that dysregulation of cuproptosis was closely related to gynecological oncology and attributed to metabolism regulation.

Next, we classified OC, CESC, and UCEC into two subtypes based on the expression of CRG network genes, respectively. We found that the DEGs were enriched in multiple metabolisms regulation in OC, CESC, and UCEC (Supplementary Figs. 2–7). Moreover, we also found that the difference between each cluster was significantly correlated with ferroptosis in these gynecological oncology patients (Supplementary Figs. 3, 5 and 7). Since previous studies indicated that ferroptosis was significantly correlated to immune infiltration in cancer^30,31^, Wang and his colleagues indicated that activated CD8^+^ T cells could enhance the level of ferroptosis in carcinogenesis^30^. Therefore, cuproptosis might induce ferroptosis and subsequently contribute to immune infiltration. In our study, we found a significant difference in other immune cell type infiltration and the expression of immune checkpoints between cluster 1 and cluster 2, which indicated that cuproptosis was significantly correlated with immune infiltration in these gynecological cancer types. However, the relationship between cuproptosis and immune infiltration has yet to be explored, which indicates that it is a direction worth exploring in detail.

We also found that CRGs had significant prognostic value in OC, CESC and UCEC (Supplementary Figs. 8–10). Song et al. found that CRGs were significantly correlated with bladder cancer patients prognosis^32^. Zhang et al. also suggested that the CRGs is helpful in liver cancer patient’s prognostic prediction and clinical treatment^33^. Bian et al. indicated that CRGs could serve as a potential prognostic predictor for clear cell renal cell carcinoma patients^34^. Bao and his colleagues confirmed that CRGs score had a significant prognostic value in lower-grade gliomas patients^35^. Sha et al. found that CRGs prognostic model was closely associated with immune infiltration in triple-negative breast cancer^36^. Taken together, these results both indicated that cuproptosis may be a broad and complicated molecular mechanism in the development of a variety of cancers, with important prognostic significance.

Moreover, we cuproptosis-related subtype presented the drug-sensitivity in different anticancer drugs, which might guide clinical therapeutics for precision medicine. A previous study has suggested that Cu ionophores exploit the massive presence of Cu in cancer tissue. Alternatively, they take advantage of cancer cell susceptibility due to oxidative stress^37^. These results show that both cuproptosis might play a key role in drug sensitivity for cancer treatment.

Then, we also further explored the prognostic significance of these CRGs in OC, CESC, and UCEC patients. Moreover, the Risk score of these prognostic models was highly correlated with different types of immune cell infiltration, indicating immune infiltration. Finally, we used a series of molecular experiments to confirm that FDX1 could impede UCEC progression by inactivating the Akt pathway.

## Conclusion

This study found cuproptosis-related subtypes in OC, CESC, and UCEC patients, suggesting a significant correlation between cuproptosis and ferroptosis. These CRGs had important prognostic significance in these gynecological cancer patients. Moreover, These CRGs, especially in FDX1, were significant in emphasizing the CD8^+^ T cell infiltration, drug sensitivity, and the immune microenvironment in CESC and UCEC.

### Supplementary Information

Supplementary Information.

## Data Availability

The data used to support the findings of this study are available from the corresponding author upon reasonable request.
