# Supplementary material for: The effect of cuproptosis-relevant genes on the immune infiltration and metabolism of gynecological oncology by multiply analysis and experiments validation
Source: Sci Rep. 2023 Nov 9;13:19474. doi: 10.1038/s41598-023-45076-5 (PMC10636103; doi:10.1038/s41598-023-45076-5)
Supplement: Supplementary file 1 — Supplementary Information. [file 41598_2023_45076_MOESM1_ESM.docx]

1. **Supplementary Data 1**

**Materials and methods**

**Data extraction.**

We extracted data for 427 cases of OSC patients, 306 cases of CESC patients, and 552 cases of UCEC patients from the TCGA database (https://portal.gdc.cancer.gov/)^1^. We also extracted data for 88 cases of ovary samples, 35 cases of endometrium samples, and 13 cases of cervical epithelium samples from the GTEX database (<https://www.gtexportal.org/>) ^2^. These data were downloaded for subsequent analysis.

**DNA alteration analyses, PPI network construction, and Function enrichment analyses.**

The DNA alteration data for CRGs were analyzed by the cBioProtal database (<http://www.cbioportal.org/>) ^3^. Please refer to our previous study on bioinformatic methods^4^. The CRGs network was constructed by GeneMANIA 3.6.0 database (<http://www.genemania.org>) ^5^. Please refer to our previous study on bioinformatic methods ^4^. DEGs or CRGs were submitted to the DAVID database (<https://david.ncifcrf.gov/>) to make GO and KEGG enrichment analyses ^6^. Please refer to our previous study on bioinformatic methods^4^.

**Immune infiltration analyses.**

For immune score analyses, we used the R software package (immuneeconv) to assess the reliable results of immune score evaluation by EPIC.

For immune checkpoints analyses, the expression values of these 8 immune-checkpoint–relevant transcripts were extracted, and the differential expression was analyzed using the ggplot2 and pheatmap R package. For the Immune cell correlation, the correlations between gene expression and the immune score were used by pheatmap package (for drawing multi-gene correlation, EPIC) or by GSVA package and estimate package (for drawing single-gene correlation, StromalScore, ImmuneScore, ESTIMATEScore, and ssGSEA). The CNV and immune infiltration analysis for FDX1 expression based on the TIMER database (<https://cistrome.shinyapps.io/timer/>) ^7^ could refer to our previous study ^4^.

**Stemness feature** **analyses.**

Use the OCLR algorithm to calculate mRNAsi constructed by Malta et al. ^8^.

**Classification of subtypes and prognostic model construction for OC, CESC, and UCEC.**

The protocol was previously described ^9^. We used the Step algorithm based on Akaike information criterion (AIC) ^10^ to construct prognostic model construction for OC, CESC, and UCEC, respectively.

**Clinical samples.**

30 OC, 35 CESC, and 32 UCEC tissues were surgically resected in The Affiliated Cancer Hospital of Xiangya School of Medicine, Central South University (Changsha, Hunan, China) from 2015 to 2022 in keeping with the ethical standards as formulated in the Helsinki Declaration, which was approved by the research ethics committee of the Central South University.

**Cell culture and transfection.**

Human endometrial cancer cells (EFE-184) were purchased from the ATCC. SC-79, FDX1 OE plasmid, and empty vector plasmids were purchased from HonourGene (Changsha, China). For specific cell culture and transfection methods, please refer to our previous study ^9^.

**Immunohistochemistry staining.**

The experiment methods refer to our previous article ^9^. FDX1 (*Proteintech*, 12592-1-AP), PCNA (Abcam, ab29), Ki-67 (Abcam, ab209847), HK2 (Abcam, ab209847), PKM (Abcam, ab15580), and CD8 (*Proteintech*, ab237709).

**The co-culture of Peripheral blood mononuclear cells (PBMCs) and UCEC cells**

First, PBMCs are isolated from peripheral blood using density gradient centrifugation. Briefly, whole blood is collected from healthy donors and mixed with an equal volume of phosphate-buffered saline (PBS). The diluted blood is then carefully layered onto Histopaque and centrifuged at 400 × g for 30 minutes. The PBMC layer is collected, washed, and resuspended in complete culture medium. Next, tumor cells are prepared and seeded into suitable culture plates or dishes. The cell density depends on the specific experimental requirements. Adherent tumor cell lines are typically grown to approximately 70-80% confluency before co-culture. After the tumor cells have reached the desired confluency, PBMCs are added to the culture plates containing the tumor cells. The PBMC to tumor cell ratio can be adjusted based on the research objectives and cell type being studied. During the co-culture, the cells are maintained in a humidified incubator at 37°C with 5% CO_2_. Finally, at the end of the co-culture period, the cells can be harvested for flow cytometry. The antibodies for flow cytometry were obtained from Abcam: CD4 (ab213215) and CD8a (ab237709).

**Western blot.**

The experiment methods refer to our previous article ^11^. FDX1 (*Proteintech*, 12592-1-AP), Akt (Abcam, ab8805), p-AKT (Abcam, ab38449), mTOR (Abcam, ab245370 at 1/2000 dilution), p-mTOR (Abcam, ab109268), and β-actin (*Proteintech*, 81115-1-RR at 1/20000 dilution).

**Cell function assay.**

For MTT assay, EdU assay, wound healing assay, transwell invasion assay, metabolism assay, and Cloneformation analysis, the experiment methods refer to our previous article ^11, 12^.

**Statistical analyses.**

All statistical analyses were performed in the R language (Version 3.6).

1. Tomczak K, Czerwinska P, Wiznerowicz M. The Cancer Genome Atlas (TCGA): an immeasurable source of knowledge. Contemp Oncol (Pozn). 2015;19:A68-77.

2. Consortium G. The Genotype-Tissue Expression (GTEx) project. Nat Genet. 2013;45:580-5.

3. Cerami E, Gao J, Dogrusoz U, et al. The cBio cancer genomics portal: an open platform for exploring multidimensional cancer genomics data. Cancer Discov. 2012;2:401-4.

4. Li Y, Zou J, Zhang Q, et al. Systemic Analysis of the DNA Replication Regulator MCM Complex in Ovarian Cancer and Its Prognostic Value. Front Oncol. 2021;11:681261.

5. Warde-Farley D, Donaldson SL, Comes O, et al. The GeneMANIA prediction server: biological network integration for gene prioritization and predicting gene function. Nucleic Acids Res. 2010;38:W214-20.

6. Dennis G, Jr., Sherman BT, Hosack DA, et al. DAVID: Database for Annotation, Visualization, and Integrated Discovery. Genome Biol. 2003;4:P3.

7. Huang da W, Sherman BT, Lempicki RA. Systematic and integrative analysis of large gene lists using DAVID bioinformatics resources. Nat Protoc. 2009;4:44-57.

8. Malta TM, Sokolov A, Gentles AJ, et al. Machine Learning Identifies Stemness Features Associated with Oncogenic Dedifferentiation. Cell. 2018;173:338-54.e15.

9. Zhang QF, Li YK, Chen CY, et al. Identification and validation of a prognostic index based on a metabolic-genomic landscape analysis of ovarian cancer. Biosci Rep. 2020.

10. Akaike H. A new look at the statistical model identification. IEEE Transactions on Automatic Control. 1974;19:716-23.

11. Li YK, Zeng T, Guan Y, et al. Validation of ESM1 Related to Ovarian Cancer and the Biological Function and Prognostic Significance. Int J Biol Sci. 2023;19:258-80.

12. Zhang J, Li Y, Fan TY, et al. Identification of bromodomain-containing proteins prognostic value and expression significance based on a genomic landscape analysis of ovarian serous cystadenocarcinoma. Front Oncol. 2022;12:1021558.

1. **Orignal data for PCR and WB**


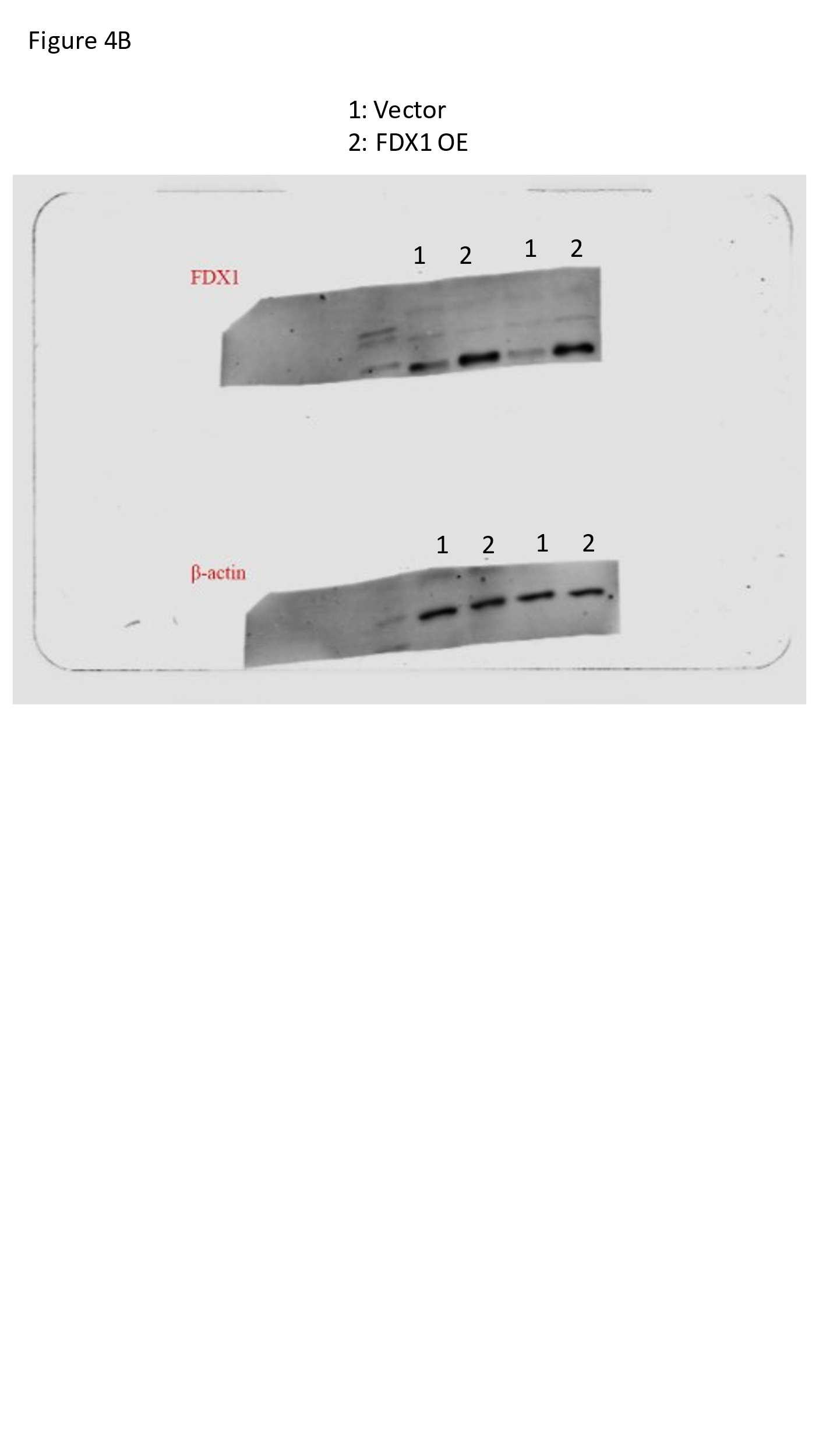


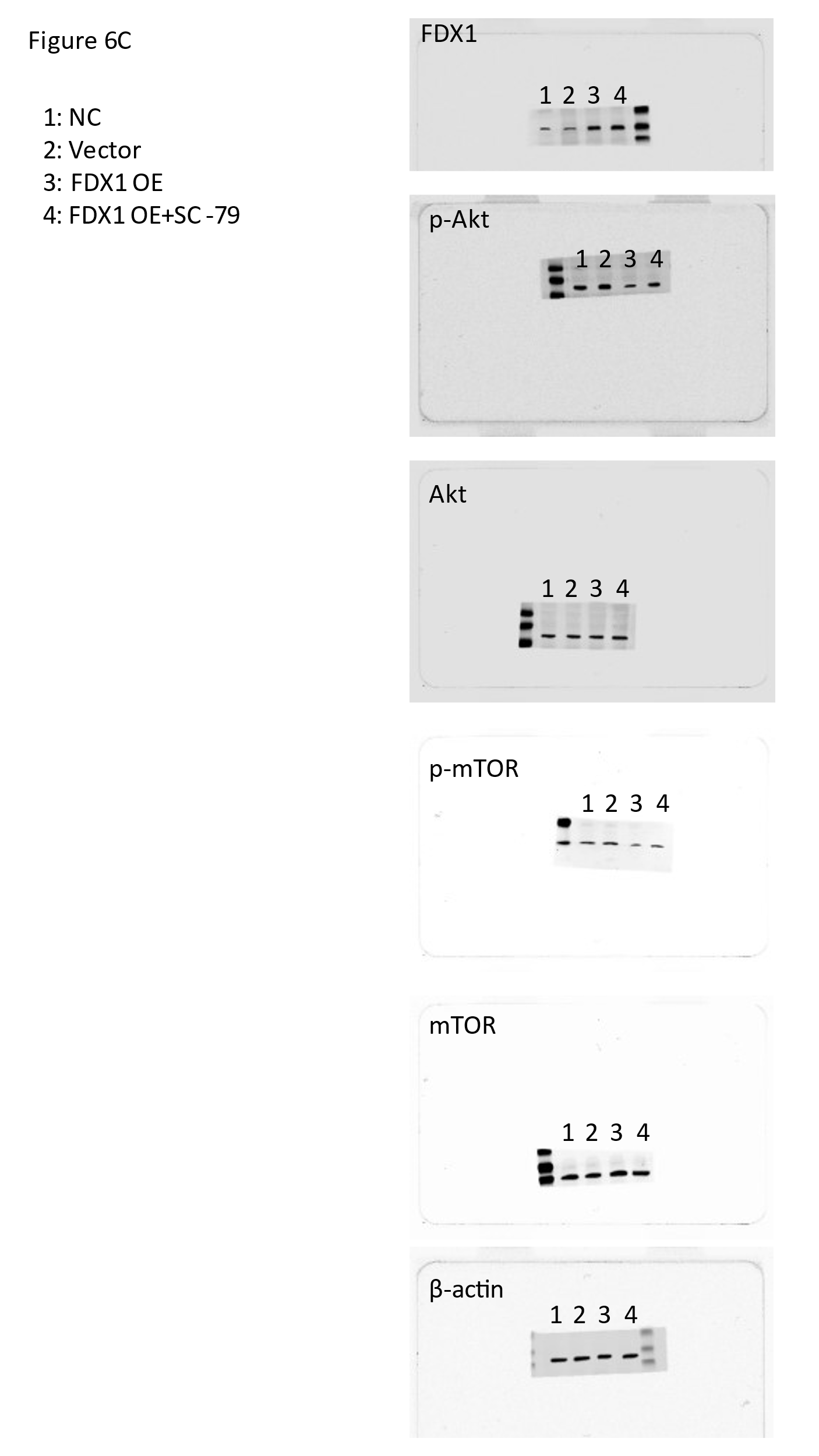


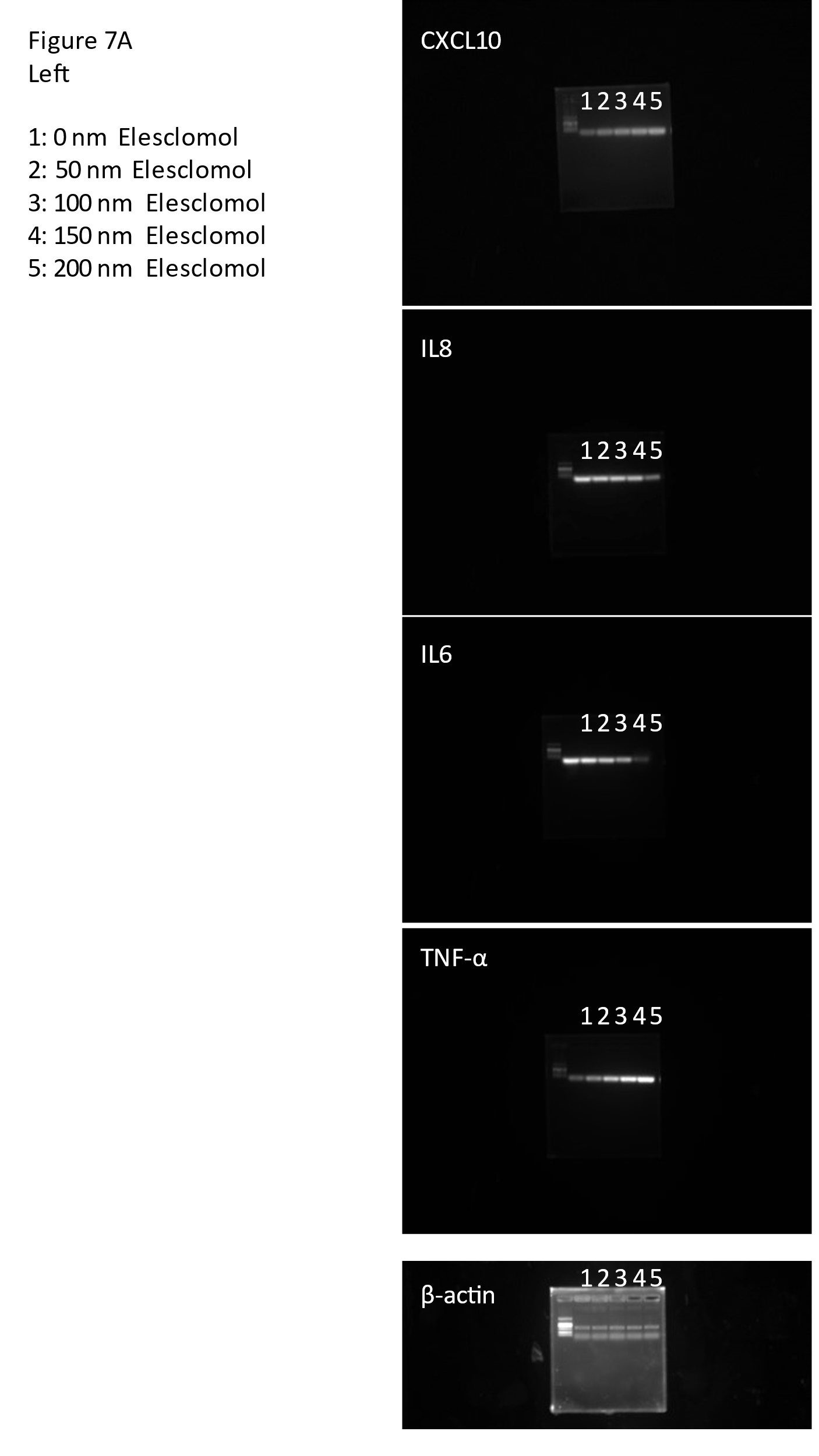


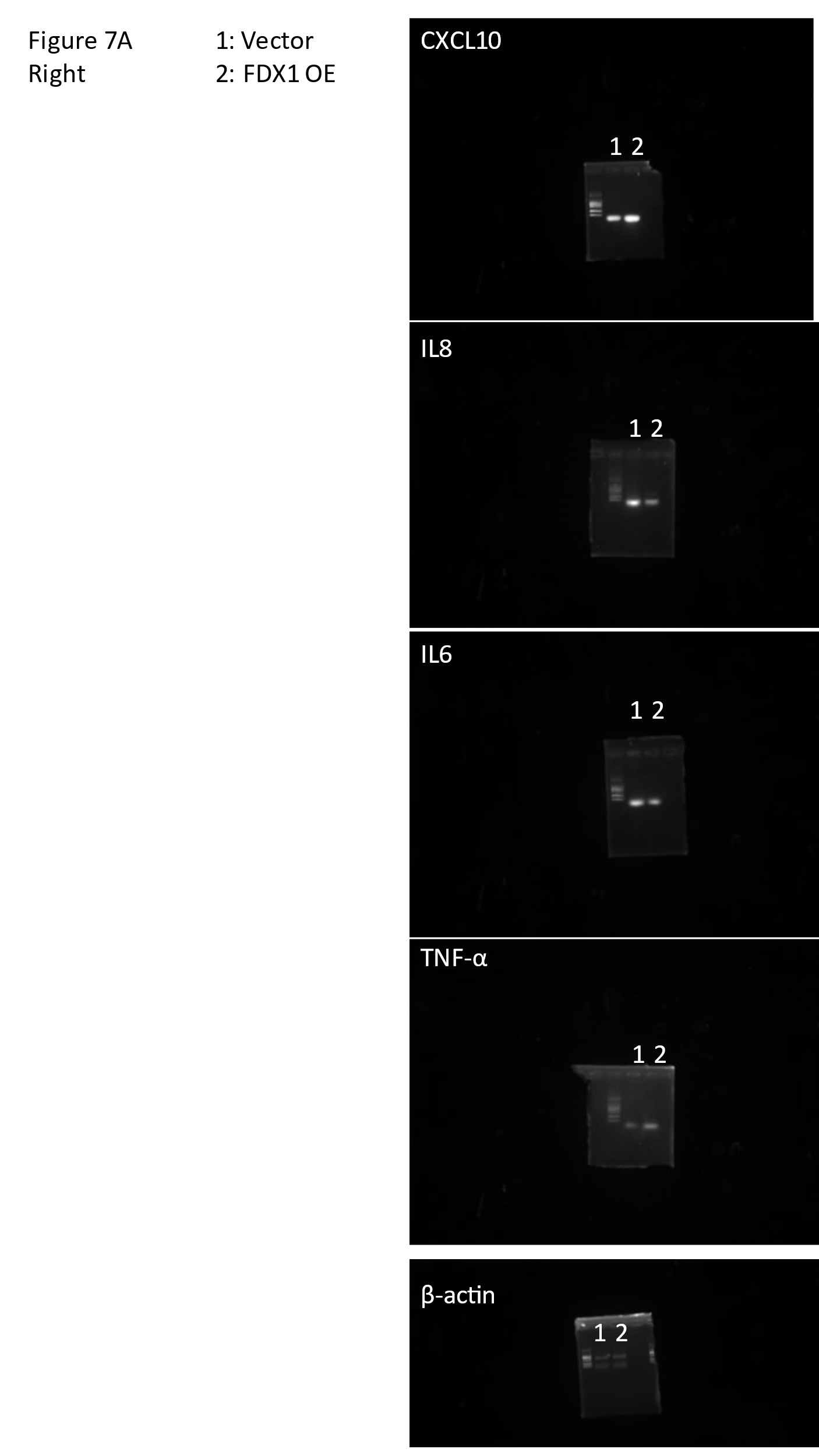


1. **Supplementary Figures and Supplementary Figure Legends:**

**
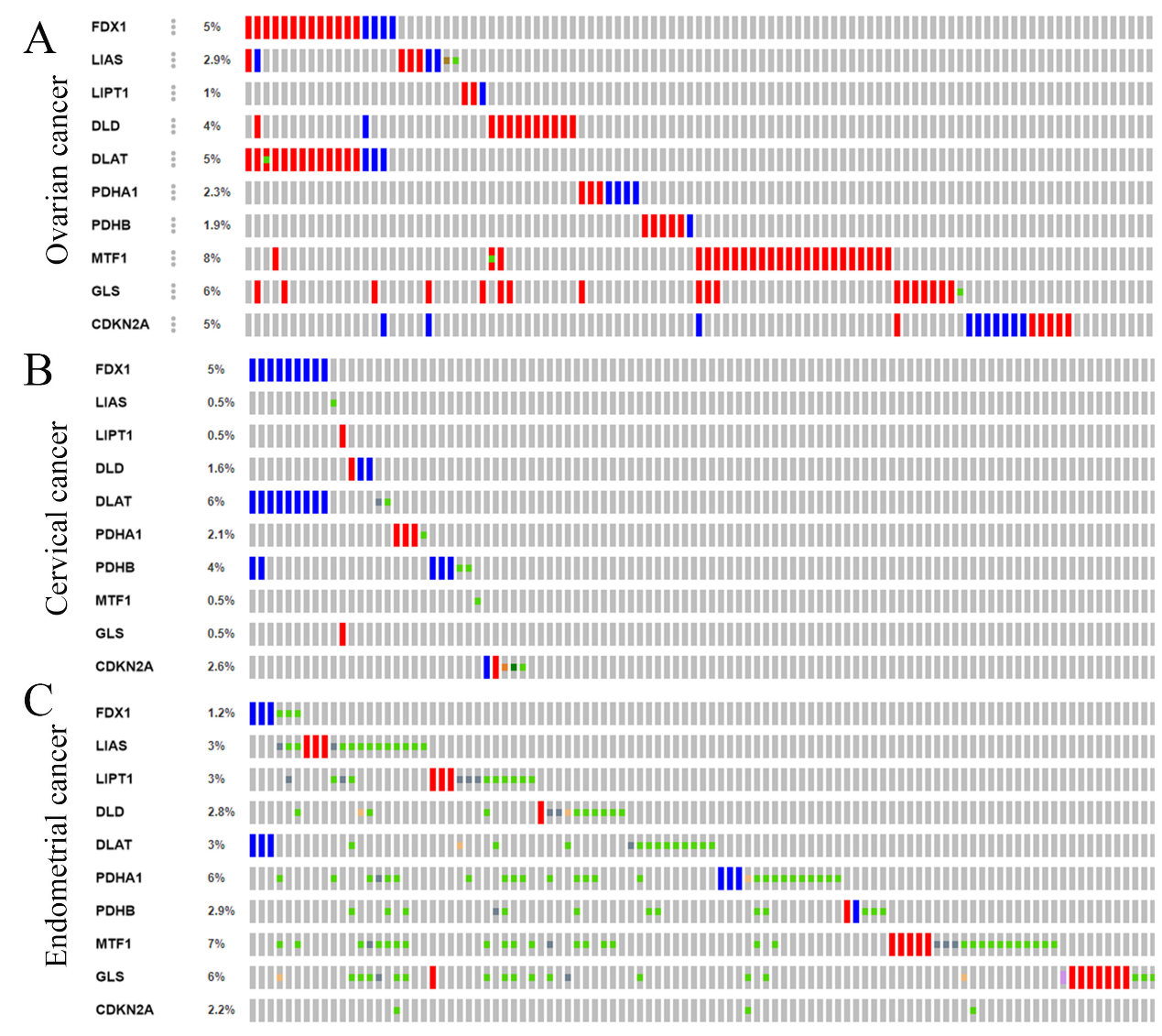
**

**Supplementary Figure 1. The DNA alteration of CRGs in different gynecological cancer types.** The DNA alteration of CRGs in A. OC, B. CESC, and C. CESC.


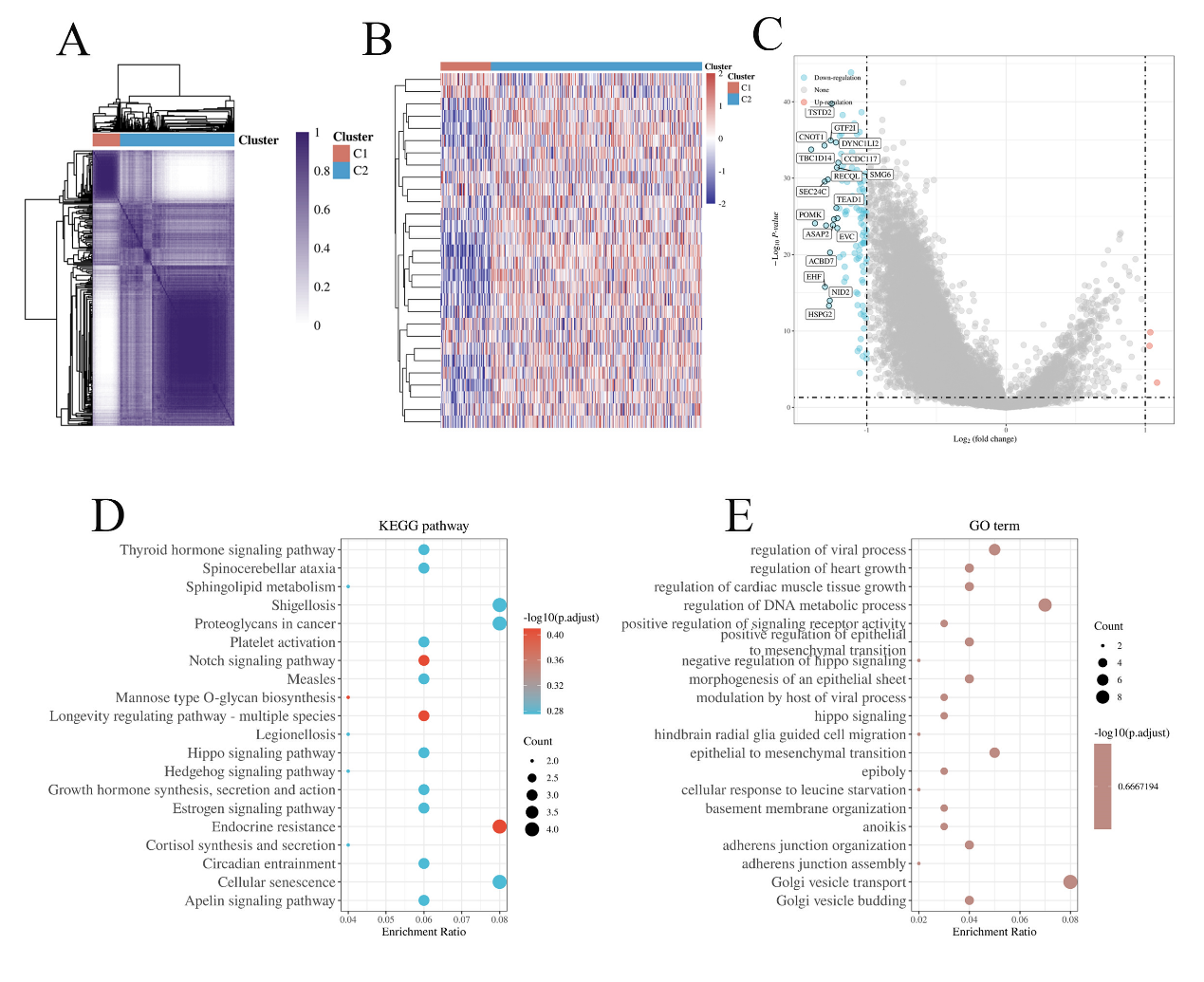


**Supplementary Figure 2. Stratification of OC by CRGs expression.** A. Consensus clustering solution by CRGs in OC patients by heatmap. B. The heatmap for DEGs level in different clusters. C. The volcano plot for DEGs in different clusters. Red indicated high level, and blue indicated low level. D. KEGG enrichment for DEGs. E. GO enrichment for DEGs.


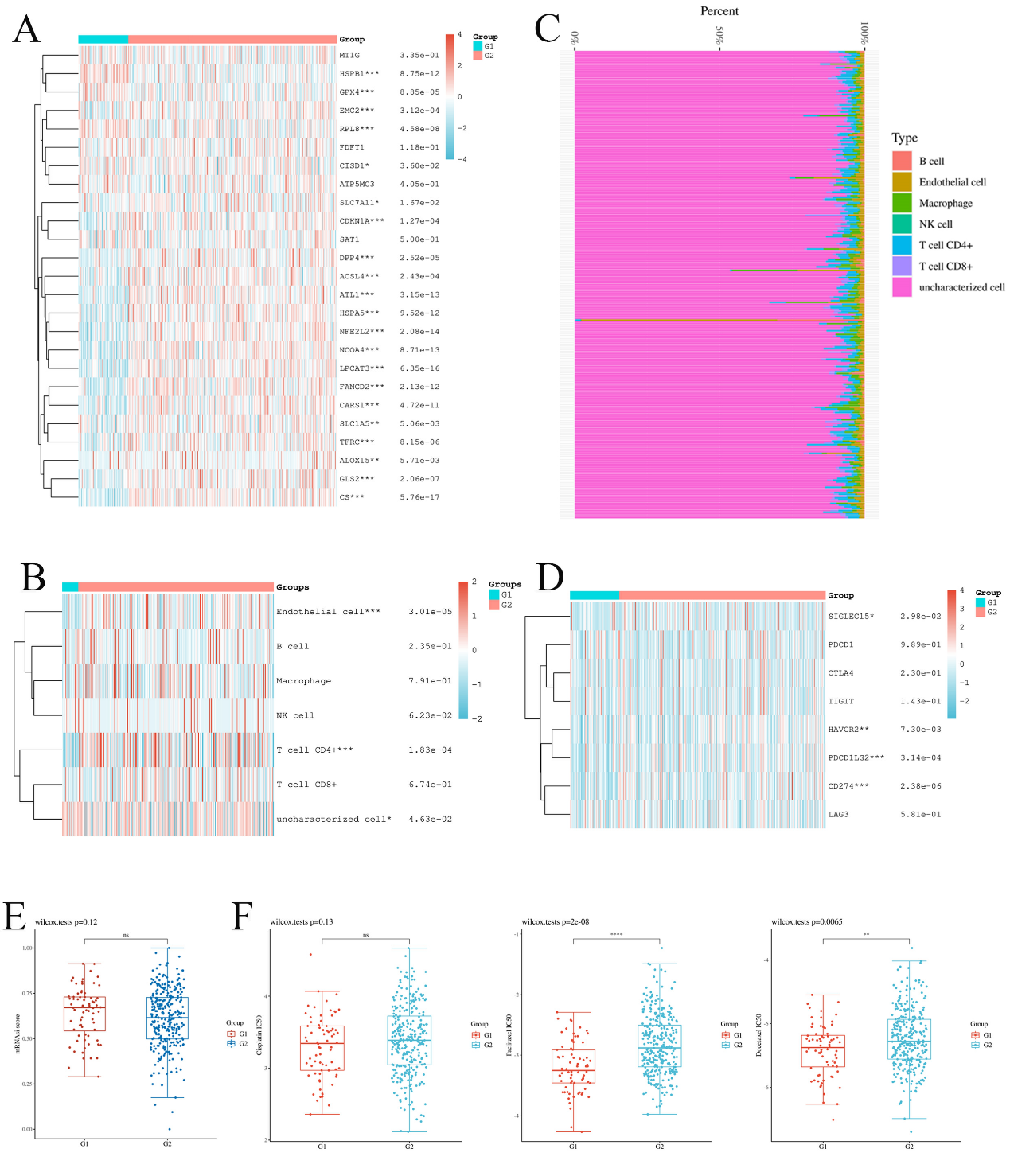


**Supplementary Figure 3. The potential functions in different OC clusters.** A. The expression of ferroptosis-related genes in different subgroups of OC by heatmap. B. Immune cell score heatmap for different immune cell expression distribution in different subgroups of OC. C. The percentage abundance of TME immune cells infiltrating in each sample. D. Heatmap for the expression of immune-checkpoint-related gene expression in different subgroups of OC. E. The stemness level in different subgroups of OC. F. The IC50 level of cisplatin, paclitaxel, and docetaxel in different subgroups of OC. ^*^ *P* < 0.05, ^**^ *P* < 0.01 and ^***^ *P* < 0.001.


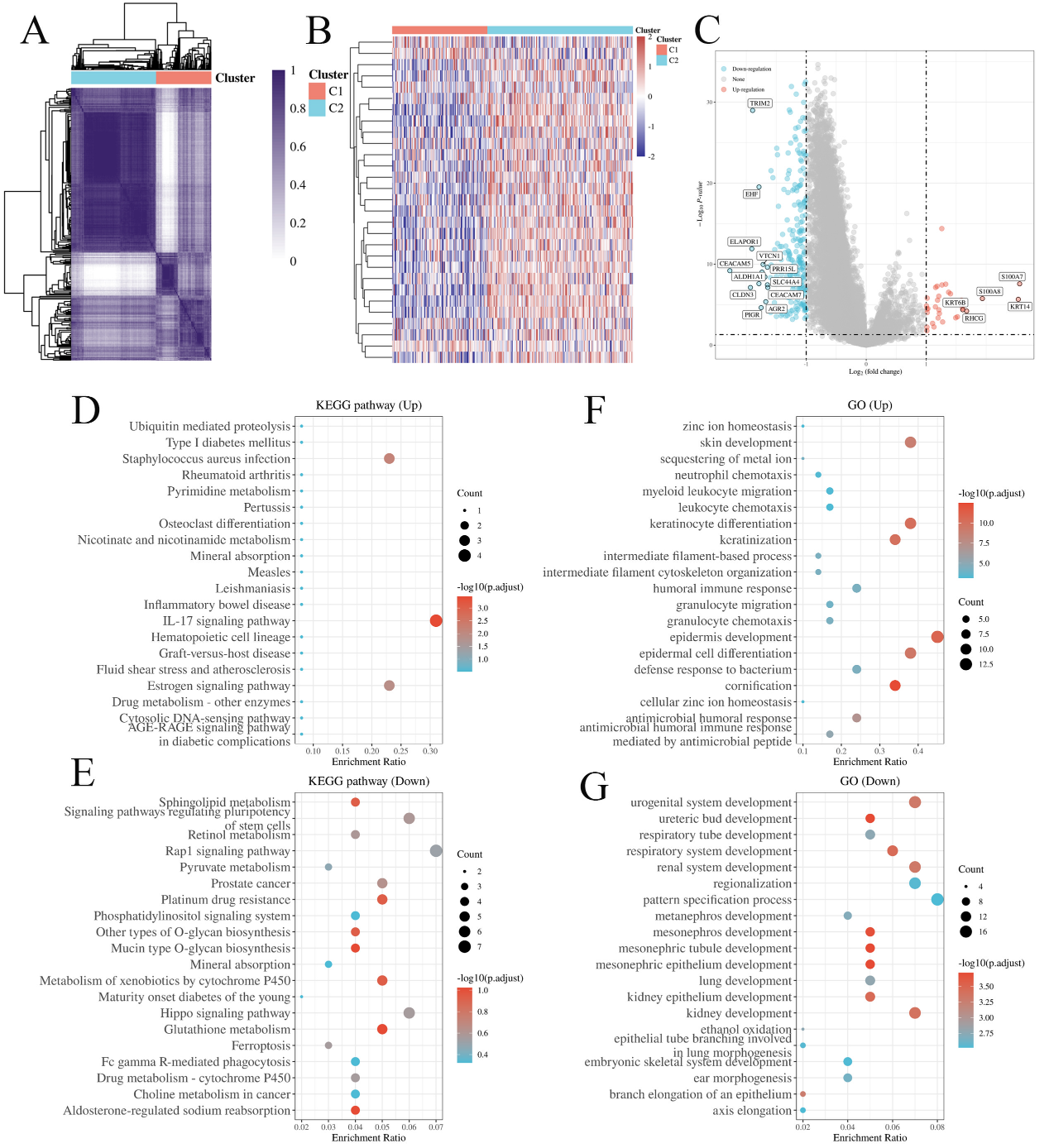


**Supplementary Figure 4. Stratification of CESC based on the expression of CRGs.** A. Heatmap depicting consensus clustering solution for CRGs in CESC patients. B. The heatmap for DEGs levels in different clusters. Red indicated a high level, and blue indicated a low level. C. The volcano plot for DEGs expression in different subgroups, red indicated high level, and blue indicated low level. KEGG enrichment for upregulated (D) and downregulated (E) DEGs. GO enrichment for upregulated (F) and downregulated (G) DEGs.


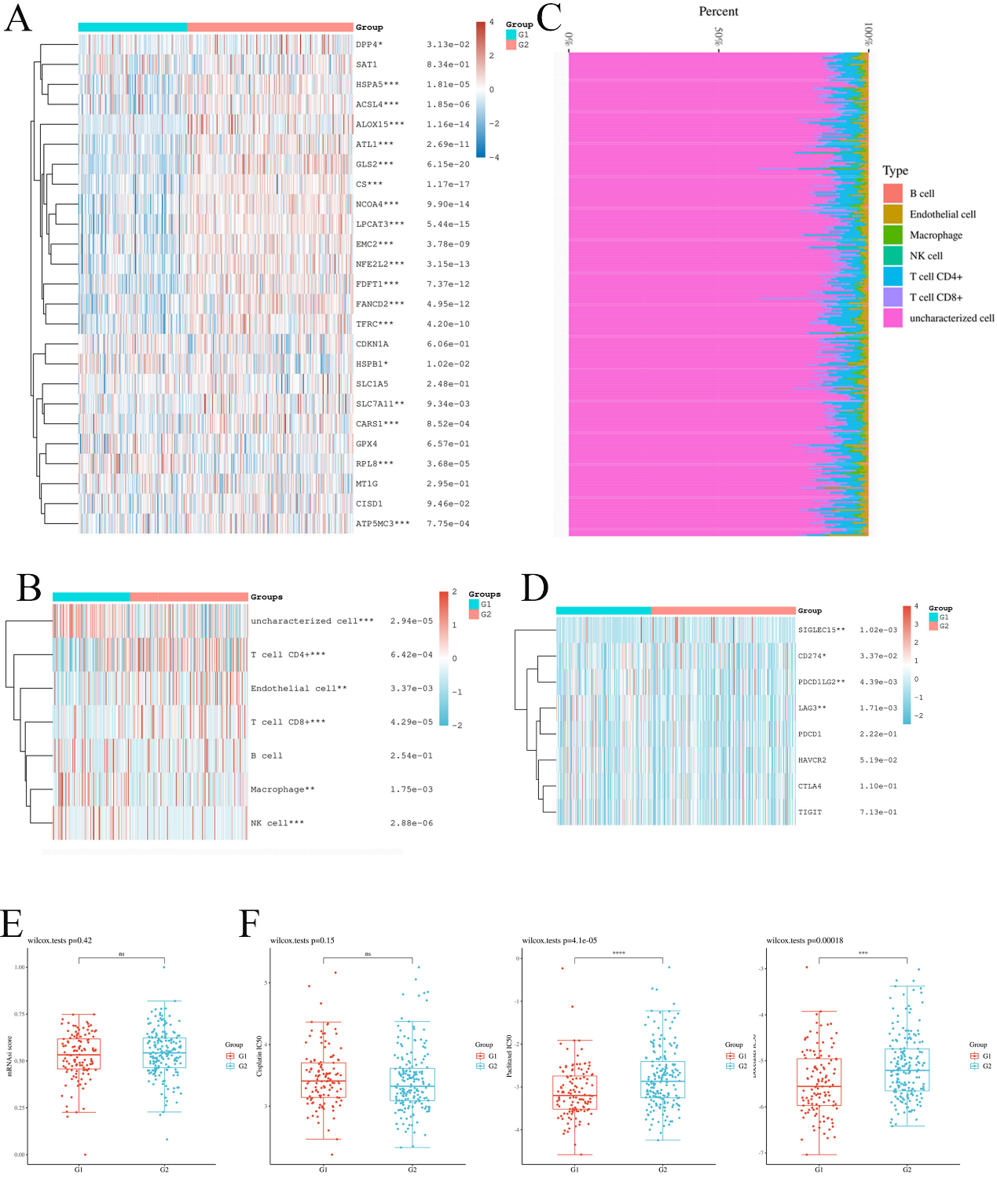


**Supplementary Figure 5. The potential functions in different subgroups of CESC.** A. Heatmap for the expression of ferroptosis-related genes in different subgroups of CESC. B. Immune cell score heatmap for different immune cell expression distribution in different subgroups of CESC. C. The percentage abundance of TME immune cells infiltrating in each sample. D. Heatmap for the expression of immune-checkpoint-related gene expression in different subgroups of CESC. E. The stemness level in different subgroups of CESC. F. The IC50 level of cisplatin, paclitaxel, and docetaxel in different subgroups of CESC. ^*^ *P* < 0.05, ^**^ *P* < 0.01 and ^***^ *P* < 0.001.


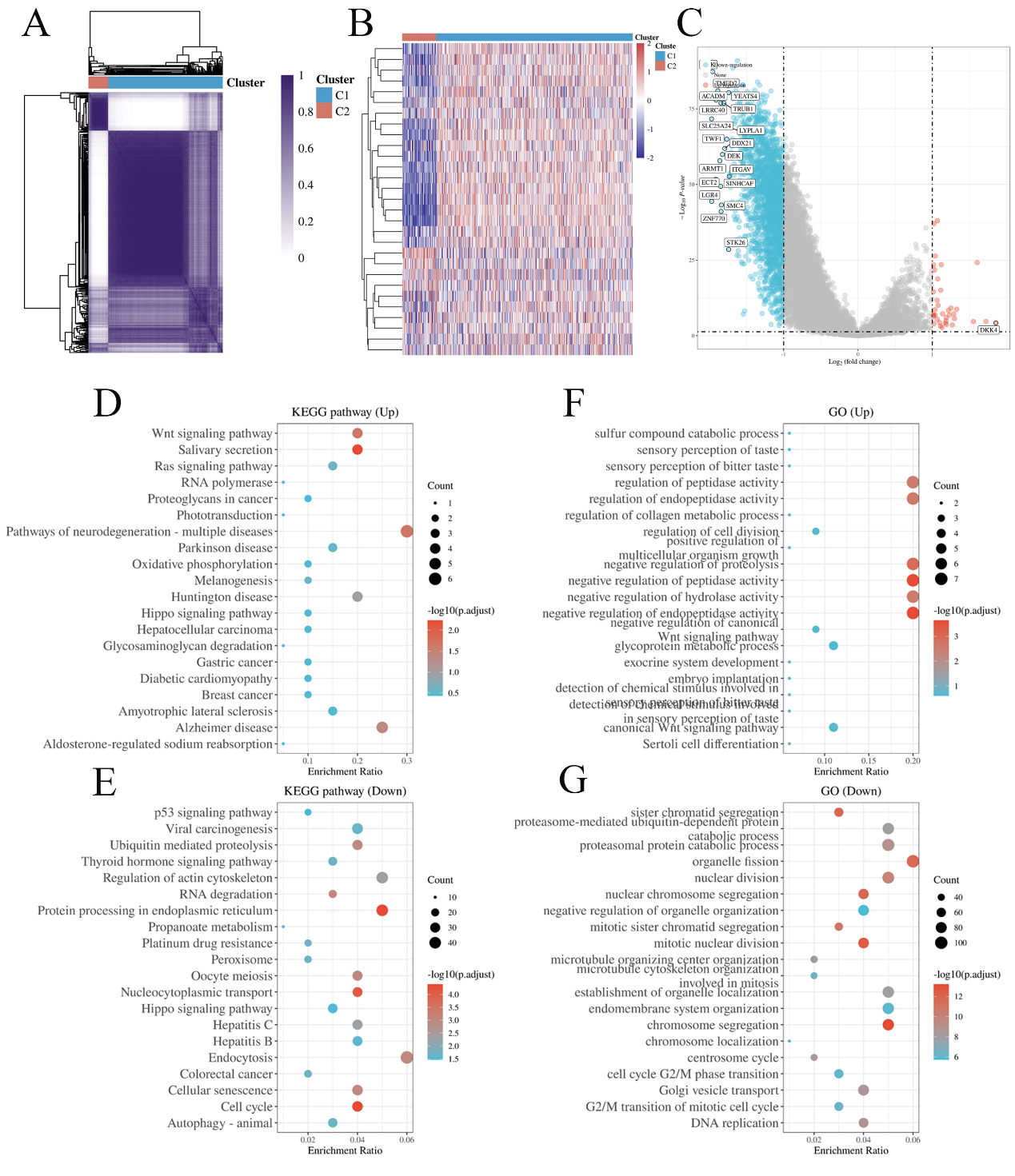


**Supplementary Figure 6. Stratification of UCEC based on CRGs expression.** A. Heatmap depicting consensus clustering solution for CRGs in UCEC patients. B. The heatmap for DEGs levels in different clusters. Red indicated a high level, and blue indicated a low level. C. The volcano plot for DEGs expression in different subgroups, red indicated high level, and blue indicated low level. KEGG enrichment for upregulated (D) and downregulated (E) DEGs. GO enrichment for upregulated (F) and downregulated (G) DEGs.


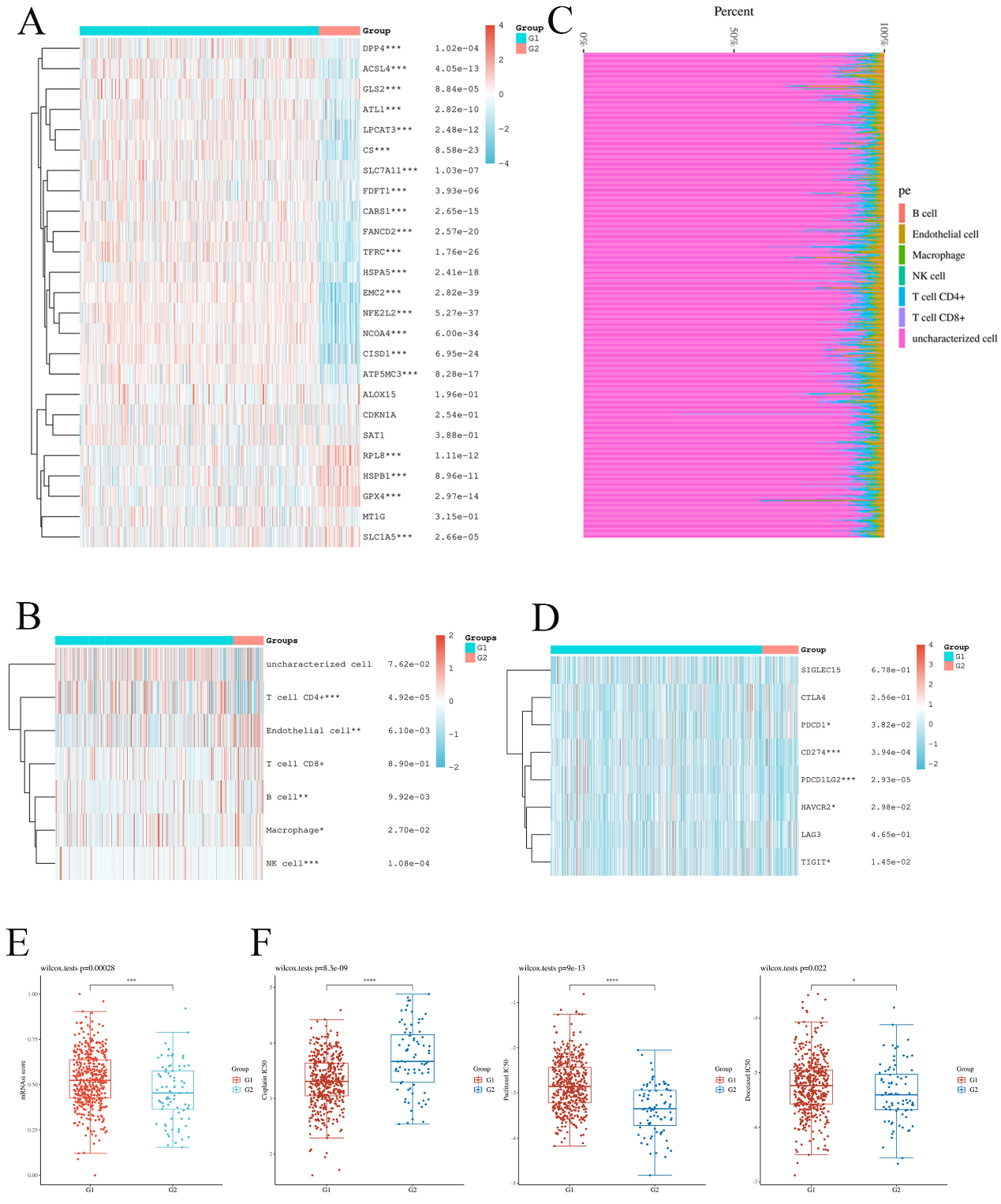


**Supplementary Figure 7. The potential functions in different subgroups of UCEC.** A. Heatmap for the expression of ferroptosis-related genes in different subgroups of UCEC. B. Immune cell score heatmap for different immune cell expression distribution in different subgroups of UCEC. C. The percentage abundance of TME immune cells infiltrating in each sample. D. Heatmap for the expression of immune-checkpoint-related gene expression in different subgroups of UCEC. E. The stemness level in different subgroups of UCEC. F. The IC50 level of cisplatin, paclitaxel, and docetaxel in different subgroups of UCEC. ^*^ *P* < 0.05, ^**^ *P* < 0.01 and ^***^ *P* < 0.001.


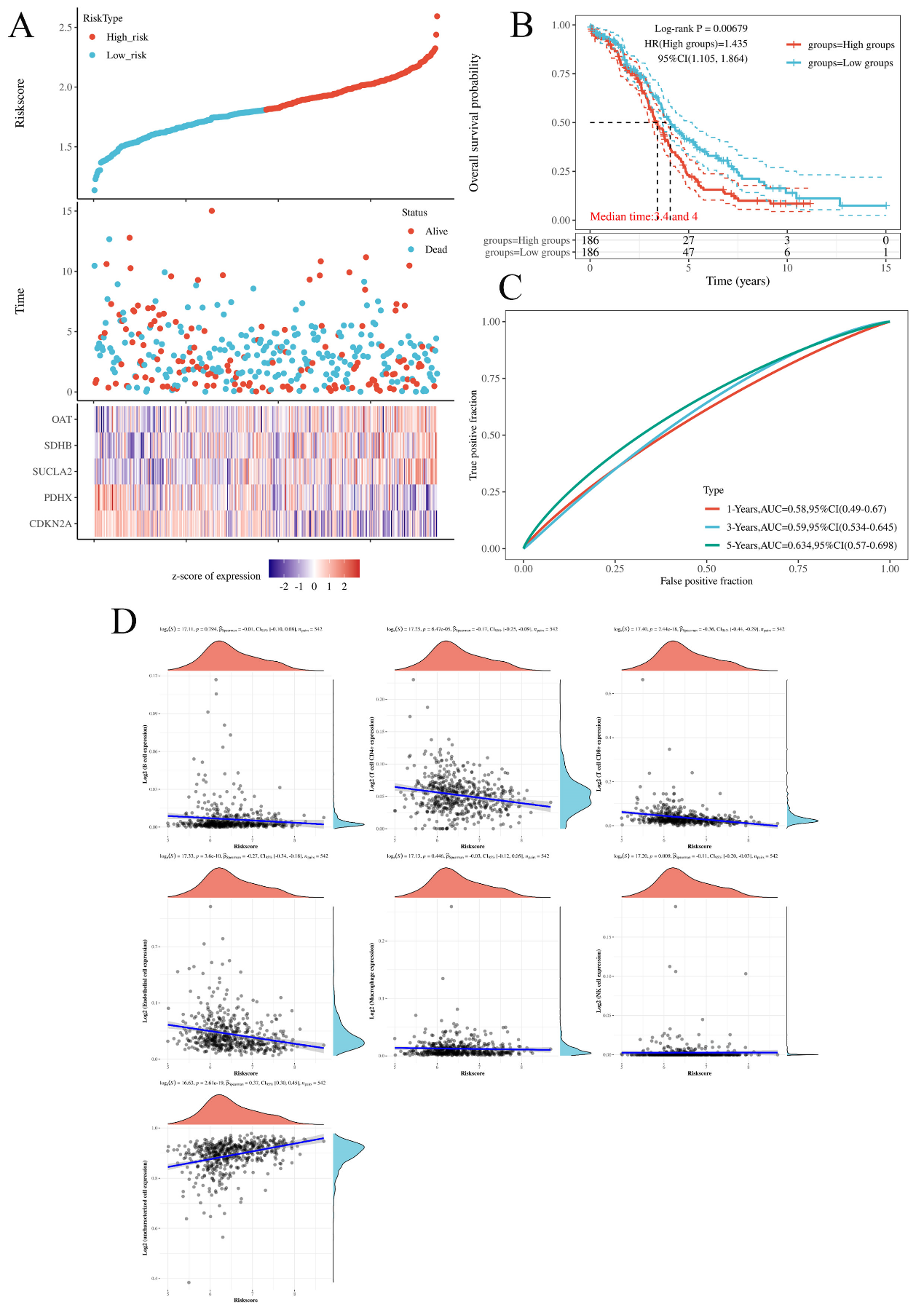


**Supplementary Figure 8. Prognostic index of OC patients.** A. The prognostic index distribution, survival, and hub gene expression profiles in OC patients. B. Survival analysis for the high- and low-risk groups of OC patients. C. AUC time-dependent ROC curves for OS in the OC patients. D. The association between Riskscore and immune infiltration was analyzed with Spearman.


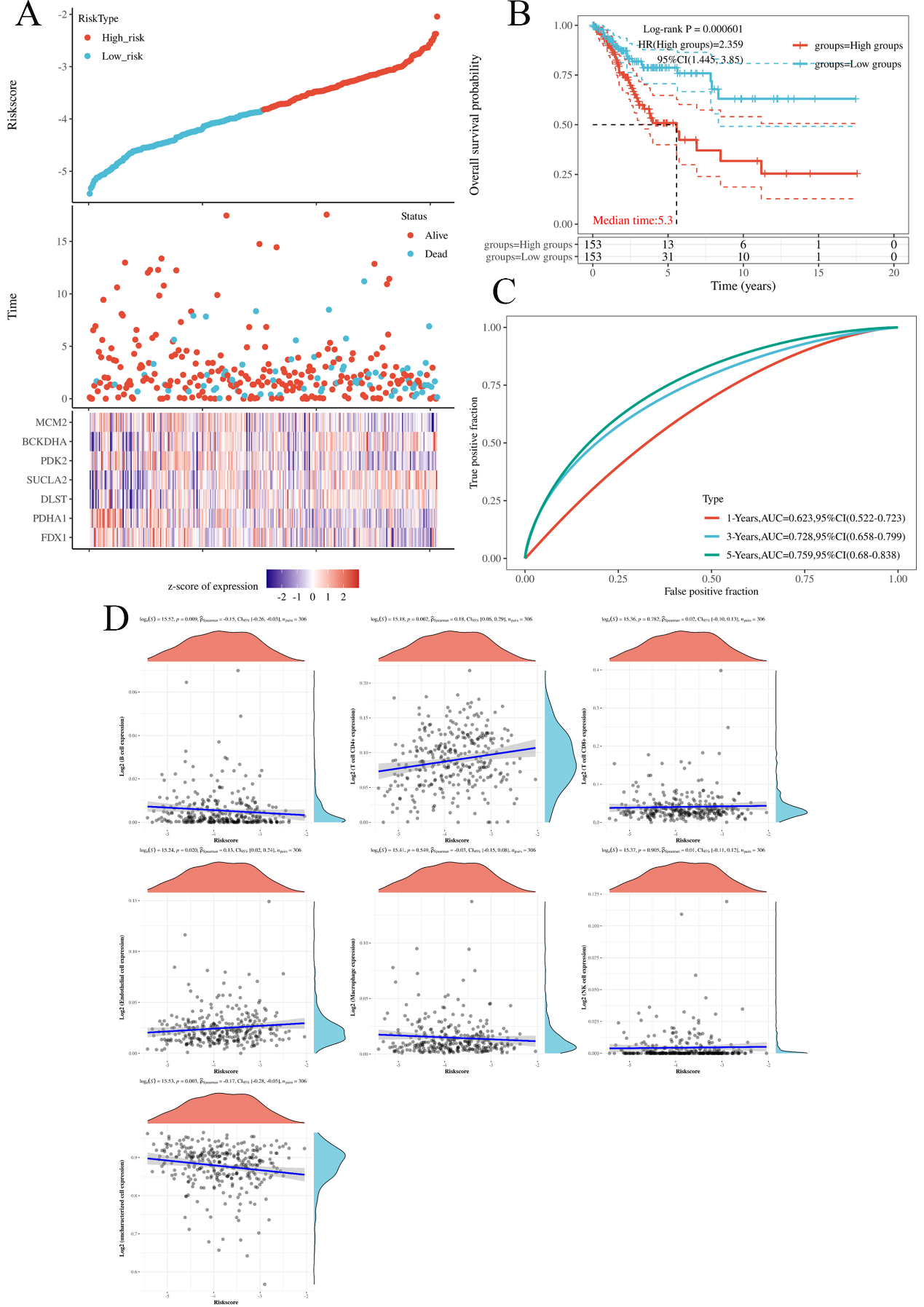


**Supplementary Figure 9. Prognostic index of CESC patients.** A. The prognostic index distribution, survival, and hub gene expression profiles in CESC patients. B. Survival analysis for the high- and low-risk groups of CESC patients. C. AUC time-dependent ROC curves for OS in the CESC patients. D. The association between Riskscore and immune infiltration was analyzed with Spearman.


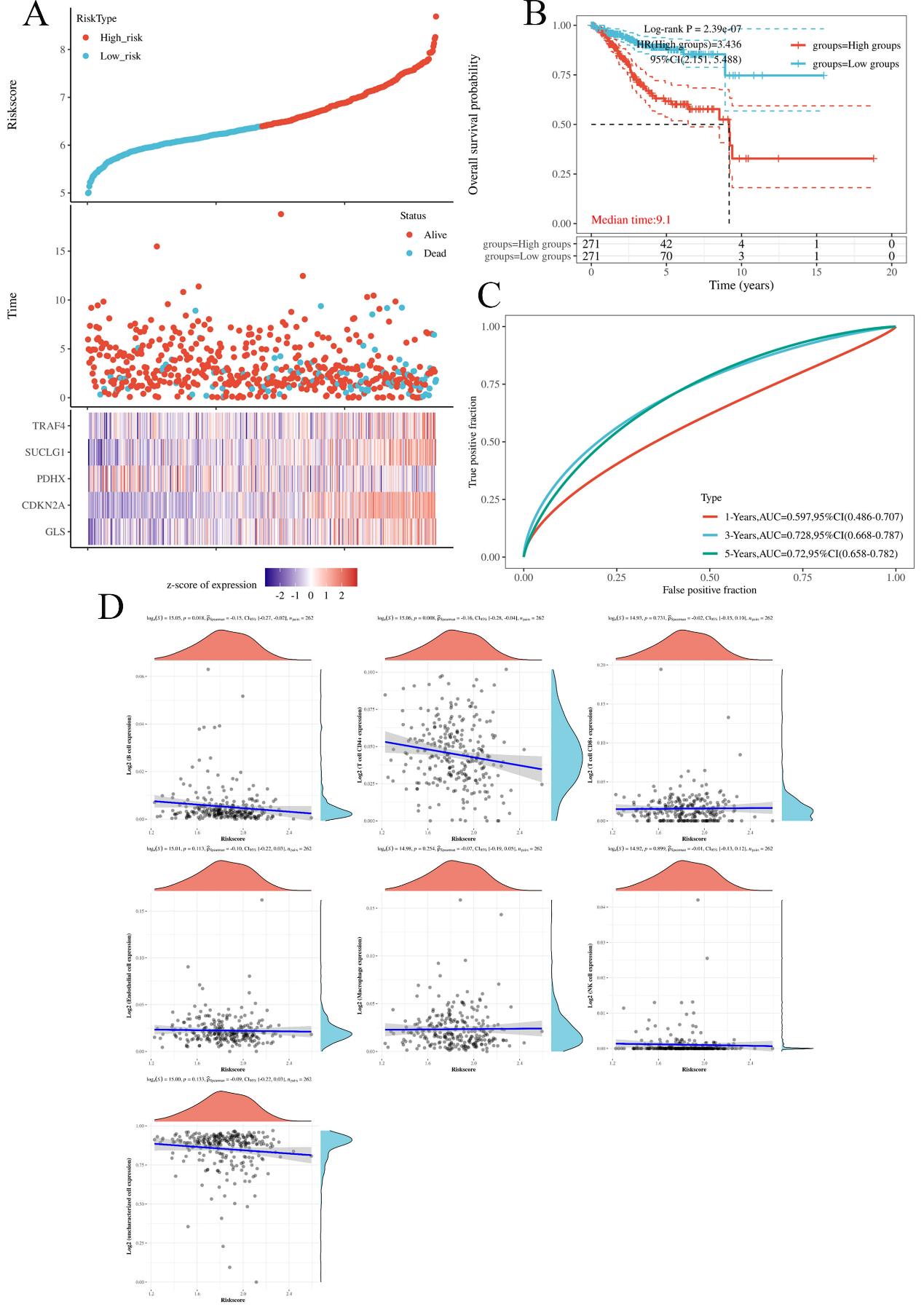


**Supplementary Figure 10. Prognostic index of UCEC patients.** A. The prognostic index distribution, survival, and hub gene expression profiles in UCEC patients. B. Survival analysis for the high- and low-risk groups of UCEC patients. C. AUC time-dependent ROC curves for OS in the UCEC patients. D. The association between Riskscore and immune infiltration was analyzed with Spearman.


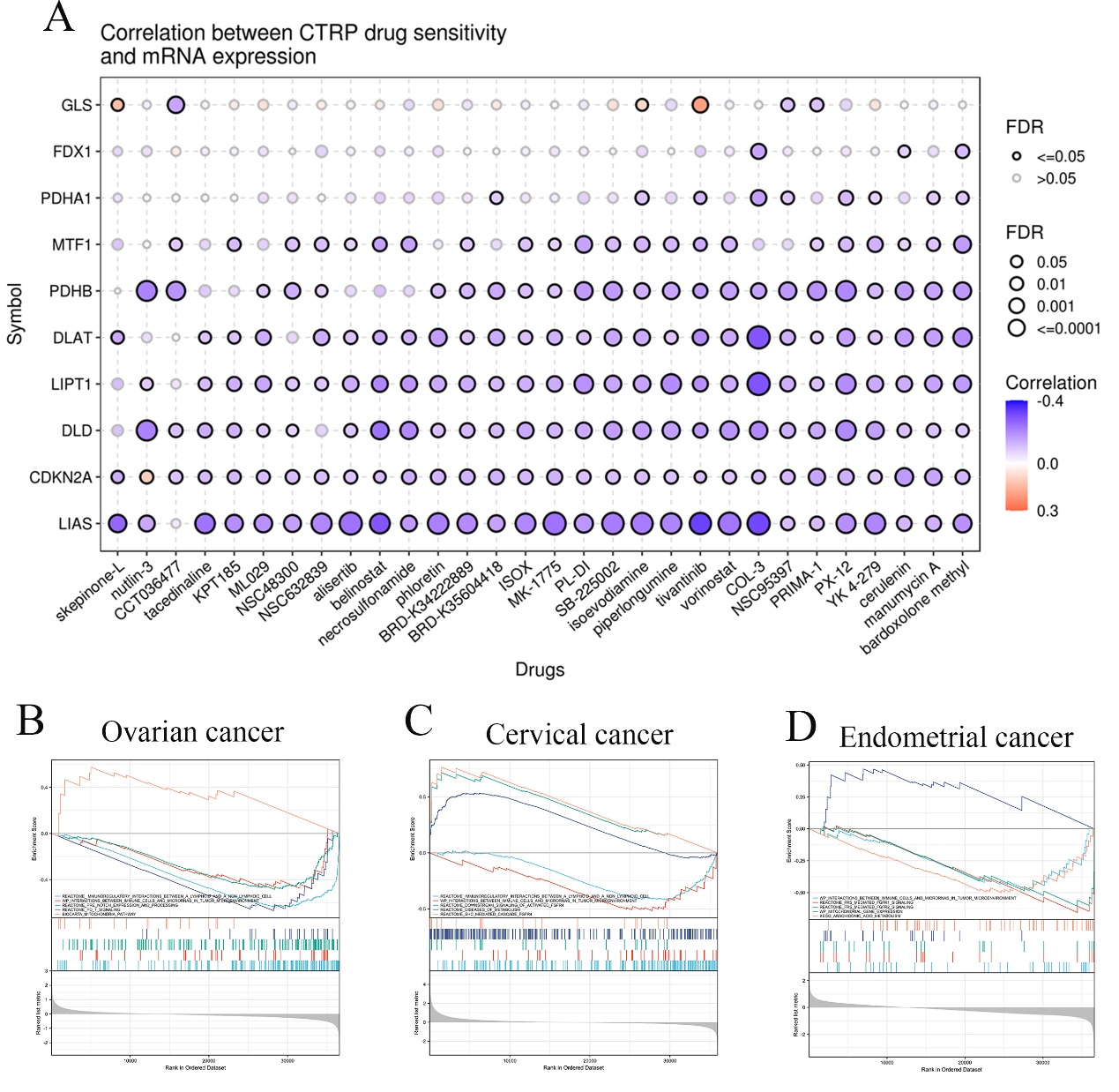


**Supplementary Figure 11. The drug sensitivity and molecular function of FDX1 in different gynecological cancer types.** A. The drug sensitivity analyses for CRGs. B. The GSEA analyses for FDX1 in OC. C. The GSEA analyses for FDX1 in CESC. D. The GSEA analyses for FDX1 in UCEC.

1. **Supplementary Tables**

**Supplementary Table 1. Characterization and correlation of FDX1 expression levels in patients with OC.**

| Characteristic | Low expression of FDX1 | High expression of FDX1 | p |
| --- | --- | --- | --- |
| n | 153 | 153 |  |
| T stage, n (%) |  |  | 0.005 |
| T1 | 59 (24.3%) | 81 (33.3%) |  |
| T2 | 39 (16%) | 33 (13.6%) |  |
| T3 | 17 (7%) | 4 (1.6%) |  |
| T4 | 4 (1.6%) | 6 (2.5%) |  |
| N stage, n (%) |  |  | 0.039 |
| N0 | 54 (27.7%) | 80 (41%) |  |
| N1 | 35 (17.9%) | 26 (13.3%) |  |
| M stage, n (%) |  |  | 0.935 |
| M0 | 56 (44.1%) | 60 (47.2%) |  |
| M1 | 6 (4.7%) | 5 (3.9%) |  |
| Clinical stage, n (%) |  |  | 0.262 |
| Stage I | 72 (24.1%) | 90 (30.1%) |  |
| Stage II | 40 (13.4%) | 29 (9.7%) |  |
| Stage III | 24 (8%) | 22 (7.4%) |  |
| Stage IV | 12 (4%) | 10 (3.3%) |  |
| Radiation therapy, n (%) |  |  | 0.726 |
| No | 63 (20.6%) | 59 (19.3%) |  |
| Yes | 90 (29.4%) | 94 (30.7%) |  |
| Primary therapy outcome, n (%) |  |  | 0.118 |
| PD | 16 (7.3%) | 7 (3.2%) |  |
| SD | 3 (1.4%) | 3 (1.4%) |  |
| PR | 2 (0.9%) | 6 (2.7%) |  |
| CR | 88 (40.2%) | 94 (42.9%) |  |
| BMI, n (%) |  |  | 0.746 |
| <=25 | 49 (18.8%) | 51 (19.6%) |  |
| >25 | 83 (31.9%) | 77 (29.6%) |  |
| Weight, n (%) |  |  | 1.000 |
| <=70 | 70 (25.3%) | 68 (24.5%) |  |
| >70 | 70 (25.3%) | 69 (24.9%) |  |
| Age, n (%) |  |  | 0.725 |
| <=50 | 92 (30.1%) | 96 (31.4%) |  |
| >50 | 61 (19.9%) | 57 (18.6%) |  |
| Histological type, n (%) |  |  | < 0.001 |
| Adenosquamous | 11 (3.6%) | 42 (13.7%) |  |
| Squamous cell carcinoma | 142 (46.4%) | 111 (36.3%) |  |
| Histologic grade, n (%) |  |  | 0.047 |
| G1 | 8 (2.9%) | 11 (4%) |  |
| G2 | 77 (28.1%) | 58 (21.2%) |  |
| G3 | 50 (18.2%) | 69 (25.2%) |  |
| G4 | 0 (0%) | 1 (0.4%) |  |
| Menopause status, n (%) |  |  | 0.652 |
| Pre | 58 (24.9%) | 68 (29.2%) |  |
| Peri | 14 (6%) | 11 (4.7%) |  |
| Post | 40 (17.2%) | 42 (18%) |  |
| Birth control pill history, n (%) |  |  | 0.127 |
| No | 52 (32.9%) | 37 (23.4%) |  |
| Yes | 31 (19.6%) | 38 (24.1%) |  |
| Keratinizing squamous cell carcinoma present, n (%) |  |  | 0.079 |
| No | 68 (22.2%) | 52 (17%) |  |
| Yes | 85 (27.8%) | 101 (33%) |  |
| Age, meidan (IQR) | 46 (38, 57) | 46 (38, 55) | 0.686 |

FDX1 is associated with age in patients with OC. P <0.05 was considered statistically significant.

**Supplementary Table 2. Characterization and correlation of FDX1 expression levels in CESC patients.**

| Characteristic | Low expression of FDX1 | High expression of FDX1 | p |
| --- | --- | --- | --- |
| n | 189 | 190 |  |
| FIGO stage, n (%) |  |  | 0.311 |
| Stage I | 0 (0%) | 1 (0.3%) |  |
| Stage II | 15 (4%) | 8 (2.1%) |  |
| Stage III | 144 (38.3%) | 151 (40.2%) |  |
| Stage IV | 27 (7.2%) | 30 (8%) |  |
| Primary therapy outcome, n (%) |  |  | 0.680 |
| PD | 15 (4.9%) | 12 (3.9%) |  |
| SD | 10 (3.2%) | 12 (3.9%) |  |
| PR | 24 (7.8%) | 19 (6.2%) |  |
| CR | 103 (33.4%) | 113 (36.7%) |  |
| Race, n (%) |  |  | 0.446 |
| Asian | 5 (1.4%) | 7 (1.9%) |  |
| Black or African American | 10 (2.7%) | 15 (4.1%) |  |
| White | 169 (46.3%) | 159 (43.6%) |  |
| Age, n (%) |  |  | 0.136 |
| <=60 | 96 (25.3%) | 112 (29.6%) |  |
| >60 | 93 (24.5%) | 78 (20.6%) |  |
| Histologic grade, n (%) |  |  | 0.322 |
| G1 | 1 (0.3%) | 0 (0%) |  |
| G2 | 26 (7%) | 19 (5.1%) |  |
| G3 | 159 (43.1%) | 163 (44.2%) |  |
| G4 | 0 (0%) | 1 (0.3%) |  |
| Anatomic neoplasm subdivision, n (%) |  |  | 0.828 |
| Unilateral | 52 (14.6%) | 50 (14%) |  |
| Bilateral | 125 (35%) | 130 (36.4%) |  |
| Venous invasion, n (%) |  |  | 0.128 |
| No | 25 (23.8%) | 16 (15.2%) |  |
| Yes | 28 (26.7%) | 36 (34.3%) |  |
| Lymphatic invasion, n (%) |  |  | 1.000 |
| No | 24 (16.1%) | 24 (16.1%) |  |
| Yes | 52 (34.9%) | 49 (32.9%) |  |
| Tumor residual, n (%) |  |  | 0.764 |
| NRD | 35 (10.4%) | 32 (9.6%) |  |
| RD | 132 (39.4%) | 136 (40.6%) |  |
| Tumor status, n (%) |  |  | 0.813 |
| Tumor free | 36 (10.7%) | 36 (10.7%) |  |
| With tumor | 126 (37.4%) | 139 (41.2%) |  |
| Age, meidan (IQR) | 60 (52, 71) | 57 (49.25, 66) | 0.026 |

FDX1 is associated with T stage, N stage, histological type and histological grade in CESC patients. P <0.05 was considered statistically significant.

**Supplementary Table 3. Characterization and correlation of FDX1 expression levels in UCEC patients.**

| Characteristic | Low expression of FDX1 | High expression of FDX1 | p |
| --- | --- | --- | --- |
| n | 276 | 276 |  |
| Clinical stage, n (%) |  |  | 0.226 |
| Stage I | 181 (32.8%) | 161 (29.2%) |  |
| Stage II | 20 (3.6%) | 31 (5.6%) |  |
| Stage III | 60 (10.9%) | 70 (12.7%) |  |
| Stage IV | 15 (2.7%) | 14 (2.5%) |  |
| Primary therapy outcome, n (%) |  |  | 0.517 |
| PD | 11 (2.3%) | 9 (1.9%) |  |
| SD | 2 (0.4%) | 4 (0.8%) |  |
| PR | 4 (0.8%) | 8 (1.7%) |  |
| CR | 227 (47.3%) | 215 (44.8%) |  |
| Age, n (%) |  |  | 0.349 |
| <=60 | 109 (19.9%) | 97 (17.7%) |  |
| >60 | 166 (30.2%) | 177 (32.2%) |  |
| BMI, n (%) |  |  | 0.524 |
| <=30 | 105 (20.2%) | 107 (20.6%) |  |
| >30 | 162 (31.2%) | 145 (27.9%) |  |
| Histological type, n (%) |  |  | 0.621 |
| Endometrioid | 210 (38%) | 200 (36.2%) |  |
| Mixed | 11 (2%) | 13 (2.4%) |  |
| Serous | 55 (10%) | 63 (11.4%) |  |
| Residual tumor, n (%) |  |  | 0.416 |
| R0 | 195 (47.2%) | 180 (43.6%) |  |
| R1 | 12 (2.9%) | 10 (2.4%) |  |
| R2 | 11 (2.7%) | 5 (1.2%) |  |
| Histologic grade, n (%) |  |  | 0.001 |
| G1 | 62 (11.5%) | 36 (6.7%) |  |
| G2 | 67 (12.4%) | 53 (9.8%) |  |
| G3 | 142 (26.2%) | 181 (33.5%) |  |
| Radiation therapy, n (%) |  |  | 0.152 |
| No | 149 (28.3%) | 130 (24.7%) |  |
| Yes | 116 (22%) | 132 (25%) |  |
| Surgical approach, n (%) |  |  | 0.017 |
| Minimally Invasive | 92 (17.4%) | 116 (21.9%) |  |
| open | 178 (33.6%) | 144 (27.2%) |  |
| Diabetes, n (%) |  |  | 0.825 |
| No | 165 (36.6%) | 163 (36.1%) |  |
| Yes | 64 (14.2%) | 59 (13.1%) |  |
| Hormones therapy, n (%) |  |  | 0.786 |
| No | 148 (43%) | 149 (43.3%) |  |
| Yes | 25 (7.3%) | 22 (6.4%) |  |
| Menopause status, n (%) |  |  | 0.756 |
| Pre | 18 (3.6%) | 17 (3.4%) |  |
| Peri | 10 (2%) | 7 (1.4%) |  |
| Post | 226 (44.7%) | 228 (45.1%) |  |
| Tumor invasion(%), n (%) |  |  | 0.417 |
| <50 | 135 (28.5%) | 124 (26.2%) |  |
| >=50 | 121 (25.5%) | 94 (19.8%) |  |
| Age, meidan (IQR) | 63 (56, 70) | 64 (58, 72) | 0.061 |

FDX1 is associated with histological grade and surgical approach in patients with UCEC. P <0.05 was considered statistically significant.
